# Rapid Intrahost Evolution of Human Cytomegalovirus Is Shaped by Demography and Positive Selection

**DOI:** 10.1371/journal.pgen.1003735

**Published:** 2013-09-26

**Authors:** Nicholas Renzette, Laura Gibson, Bornali Bhattacharjee, Donna Fisher, Mark R. Schleiss, Jeffrey D. Jensen, Timothy F. Kowalik

**Affiliations:** 1 Department of Microbiology and Physiological Systems, University of Massachusetts Medical School, Worcester, Massachusetts, United States of America; 2 Departments of Pediatrics and Medicine, Divisions of Infectious Diseases and Immunology, University of Massachusetts Medical School, Worcester, Massachusetts, United States of America; 3 Department of Pediatrics, Division of Pediatric Infectious Diseases, Baystate Children's Hospital, Tufts University School of Medicine, Springfield, Massachusetts, United States of America; 4 Center for Infectious Diseases and Microbiology Translational Research, Division of Pediatric Infectious Diseases and Department of Pediatrics, University of Minnesota Medical School, Minneapolis, Minnesota, United States of America; 5 School of Life Sciences, Ecole Polytechnique Fédérale de Lausanne (EPFL), Lausanne, Switzerland; 6 Swiss Institute of Bioinformatics (SIB), Lausanne, Switzerland; 7 Immunology and Virology Program, University of Massachusetts Medical School, Worcester, Massachusetts, United States of America; University of Arizona, United States of America

## Abstract

Populations of human cytomegalovirus (HCMV), a large DNA virus, are highly polymorphic in patient samples, which may allow for rapid evolution within human hosts. To understand HCMV evolution, longitudinally sampled genomic populations from the urine and plasma of 5 infants with symptomatic congenital HCMV infection were analyzed. Temporal and compartmental variability of viral populations were quantified using high throughput sequencing and population genetics approaches. HCMV populations were generally stable over time, with ∼88% of SNPs displaying similar frequencies. However, samples collected from plasma and urine of the same patient at the same time were highly differentiated with approximately 1700 consensus sequence SNPs (1.2% of the genome) identified between compartments. This inter-compartment differentiation was comparable to the differentiation observed in unrelated hosts. Models of demography (i.e., changes in population size and structure) and positive selection were evaluated to explain the observed patterns of variation. Evidence for strong bottlenecks (>90% reduction in viral population size) was consistent among all patients. From the timing of the bottlenecks, we conclude that fetal infection occurred between 13–18 weeks gestational age in patients analyzed, while colonization of the urine compartment followed roughly 2 months later. The timing of these bottlenecks is consistent with the clinical histories of congenital HCMV infections. We next inferred that positive selection plays a small but measurable role in viral evolution within a single compartment. However, positive selection appears to be a strong and pervasive driver of evolution associated with compartmentalization, affecting ≥34 of the 167 open reading frames (∼20%) of the genome. This work offers the most detailed map of HCMV *in vivo* evolution to date and provides evidence that viral populations can be stable or rapidly differentiate, depending on host environment. The application of population genetic methods to these data provides clinically useful information, such as the timing of infection and compartment colonization.

## Author Summary

The large, dsDNA virus Human cytomegalovirus (HCMV) is the most genetically complex viral pathogen of humans. HCMV populations are highly variable, which may allow the virus to evolve in human hosts on short timescales. We tested this hypothesis by longitudinally sampling HCMV populations from the urine and/or plasma of congenitally infected infants. We found that HCMV is generally stable within a compartment, but rapidly evolves when crossing host compartments. In fact, HCMV sampled from two compartments of the same host is as different as HCMV collected from unrelated hosts. We used mathematical modeling and population genetic analysis to show that both a bottleneck (i.e., a reduction in population size) associated with compartment colonization as well as positive selection are necessary to explain the observed differences between compartments. We also conclude from these data that fetal infection in these patients occurred between 13–18 weeks gestational age, consistent with the timing of symptomatic congenital HCMV infections. This study is the most detailed investigation of DNA virus evolution in human hosts to date, provides a framework for the study of other viral infections using similar techniques, and will aid in the development of new antiviral therapies and vaccines.

## Introduction

Human cytomegalovirus (HCMV) is a β-herpesvirus with seroprevalence of 30–90% in the United States [1]. In healthy individuals, primary HCMV infection is usually asymptomatic or causes a mild febrile illness. However, in the immuno-compromised or immune-naïve host, HCMV infection can lead to more severe outcomes, such as graft rejection or death. In particular, an estimated 0.7% of live-born infants per year (30,000 in the U.S.) are diagnosed with congenital CMV infection, and nearly 20% exhibit permanent neurologic sequelae [2].

HCMV has been shown to be highly polymorphic both among and within human hosts [3]–[6], and previous studies have used inter-host or inter-species divergence data to infer the evolutionary path of HCMV (and other herpesviruses) over long timescales (thousands to millions of years) [7]–[10]. These studies have shown that the virus is well-adapted to its natural host, using an impressive array of immune evasion and modulation genes to establish life-long infections [10], [11]. However, the possibility that the virus evolves within human hosts on much shorter timescales has been poorly studied. Importantly, it has recently been shown that HCMV genomic and genetic populations are highly polymorphic, with levels of intrahost diversity comparable to those observed in RNA virus populations [5], [6]. Because variation is the substrate upon which selection can act, it is possible that intrahost variation of HCMV populations allows for rapid evolution within the human host across relatively short timescales.

To better understand this process, we used high throughput sequencing to sample HCMV genomic populations from the urine and plasma of 5 infants with symptomatic congenital HCMV infection at multiple time points during the first year of age. This approach allowed us to monitor genome-wide evolution of the virus and to determine the mechanisms that contribute to pathogen evolution. We find that HCMV populations can evolve slowly within the same tissue compartment, or rapidly when moving between compartments. In order to characterize the mode and tempo of this differentiation, we constructed detailed maps of the *in vivo* demographic history of HCMV populations, and tested for signatures of positive selection. In total, this work provides the most complete insight to date into the evolutionary paths of a DNA virus as well as the mechanisms that guide these routes in human hosts.

## Results

Our previous work showed significant variability within HCMV populations sampled from the urine of congenitally infected neonates - variation that could potentially enable rapid evolution of the viral population [6]. To study the evolution of HCMV in human hosts, HCMV genomic populations were examined within fluid specimens from 5 infants with symptomatic congenital infection. Specimens were longitudinally collected from the urine (patients B101, MS1 and MS2), plasma (patient M103) or both the urine and plasma (patient B103) during the first year after birth (14 specimens in total) (Table 1). Patients B101, M103 and B103 are unrelated, while patients MS1 and MS2 are monozygotic, monochorionic twins. A previously described high throughput sequencing strategy [6] was used to study the viral populations. Briefly, HCMV genomic DNA was specifically amplified through a series of PCR reactions, the amplified DNA pools were sequenced, and the sequence output was analyzed for variants within and among viral populations. Due to improvements in high throughput sequencing technology since our initial report, the frequencies at which variants are detected has improved from ≥1.9% [6] to ≥1.02% and the error rate after filtering has improved from 6.7% [6] to 5.0% (Figure S1). An average of 854 megabases of sequence information was produced for each of the 14 specimens (Table S1). The sequence data covered an average of 85% of the genome to an average depth of 1921 genome equivalents (Table S1). The high depth and coverage allows for quantitative description of the evolution of the HCMV in vivo populations.

**Table 1:** Patient and specimen information.

Patient^1^^,^ 2	Specimen Source	Age at Specimen Collection (months or as noted)	Viral Load (copies/mL)
B101	Urine	7	1.3E+07
B101	Urine	10	1.8E+07
B103	Urine	1 week	6.7E+06
B103	Urine	6	2.6E+07
B103	Plasma	1 week	6.9E+04
B103	Plasma	6	4.9E+04
M103	Plasma	1.5	1.8E+04
M103	Plasma	5	3.9E+04
MS1	Urine	1	5.0E+07
MS1	Urine	2	6.4E+06
MS1	Urine	11	4.0E+05
MS2	Urine	1	3.7E+07
MS2	Urine	2	1.0E+07
MS2	Urine	11	4.5E+05

1 All specimens were collected from patients with confirmed congenital HCMV infections.

2 Patients MS1 and MS2 are monozygotic, monochorionic twins.

**Figure 1:**
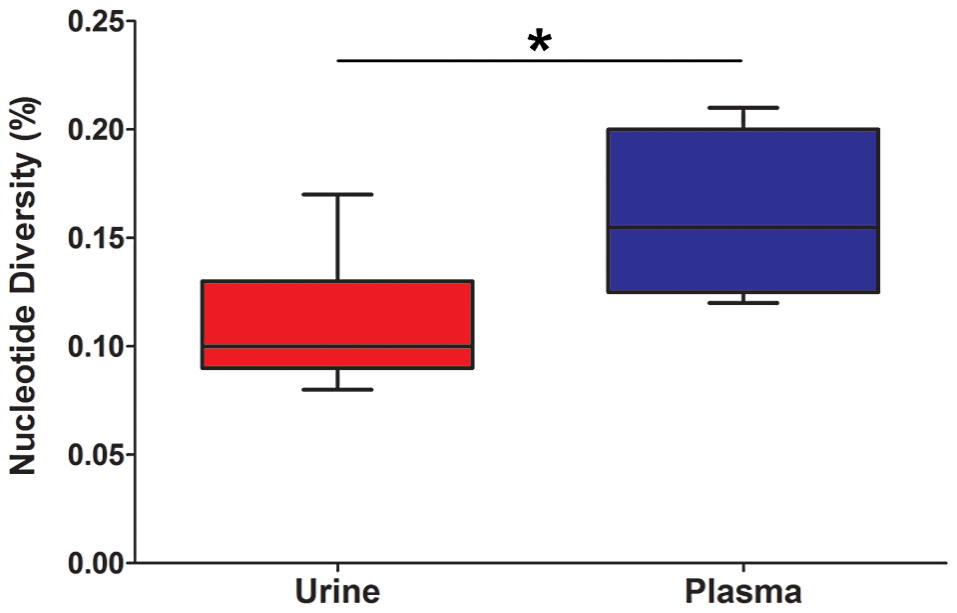
HCMV population diversity is correlated with host compartment. Box and whisker plots of the nucleotide diversity of HCMV populations sampled from the urine and plasma compartments. The boxes mark the interquartile values and the whiskers show the minimum and maximum values. The distributions are significantly different (*P* = 0.046, Mann-Whitney *U*, denoted by *), with urine populations exhibiting lower diversity values than plasma populations.

Initially, the populations were studied separately by quantifying the intrahost variation of each population. Consistent with previous work, thousands of single nucleotide polymorphisms (SNPs) were observed in each population (Table 2). On average, ∼5,400 SNPs were identified per population, which accounts for ∼3% of the positions in the viral genomes sequenced. Similar levels of variability existed at both the nucleotide and amino acid levels. To quantify the variability of the populations, nucleotide diversity values were estimated from the sequence data. These values were comparable to previous work [6], with a mean nucleotide diversity value of 0.12% across all populations. There was a negative correlation between nucleotide diversity of the viral population and time of sample collection, though the correlation was not significant (data not shown). However, a significant correlation was found between nucleotide diversity and the compartment from which the population was sampled (*P* = 0.046, Mann-Whitney), with urine populations exhibiting lower diversity levels than plasma populations (Figure 1).

**Table 2:** Genome wide summary statistics for HCMV populations.

Patient	Sample Source	Time of Collection	SNPs	Π^1^	Nonsynonymous SNPs	Π_Non_ 2	Π_Syn_ 2
B101	Urine	7 months	6,765	0.17%	4,280	0.11%	0.04%
B101	Urine	10 months	5,019	0.13%	3,153	0.07%	0.02%
B103	Urine	1 week	6,350	0.10%	3,736	0.05%	0.02%
B103	Urine	6 months	6,723	0.10%	3,776	0.05%	0.02%
B103	Plasma	1 week	4,890	0.21%	2,606	0.09%	0.09%
B103	Plasma	6 months	5,735	0.17%	3,181	0.09%	0.04%
M103	Plasma	1.5 months	3,503	0.14%	2,272	0.06%	0.04%
M103	Plasma	5 months	3,820	0.12%	2,308	0.07%	0.03%
MS1	Urine	1 month	5,049	0.09%	2,580	0.04%	0.02%
MS1	Urine	2 months	4,693	0.09%	2,475	0.04%	0.02%
MS1	Urine	11 months	5,515	0.13%	2,978	0.04%	0.03%
MS2	Urine	1 month	4,662	0.09%	2,590	0.04%	0.02%
MS2	Urine	2 months	3,950	0.08%	2,008	0.03%	0.02%
MS2	Urine	11 months	8,789	0.13%	4,870	0.06%	0.03%

1 Π is nucleotide diversity as calculated using the formula of Nei and Li [45].

2 Π_Non_ is amino acid diversity and Π_Syn_ is the diversity of synonymous SNPs. Both were calculated in the same way as Π but only using nonsynonymous or synonymous SNPs, respectively.

**Figure 2:**
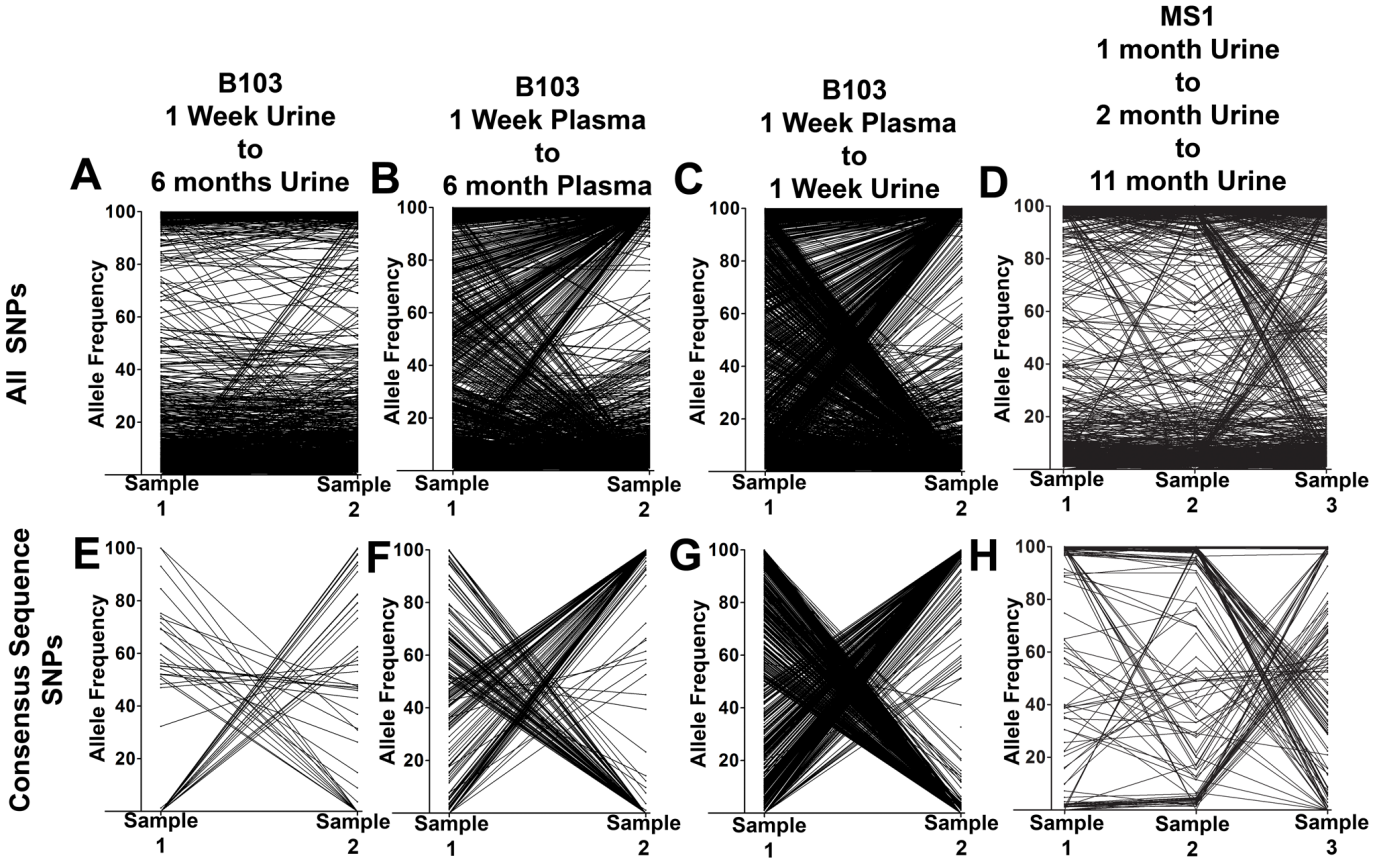
HCMV populations show patterns of both stability and differentiation. Host-matched specimens were paired either across time or compartments. The frequency of SNPs was tracked across the pairings. The trajectories show that HCMV populations can be stable, with the majority of SNPs remaining at nearly the same frequencies over time. A minority of SNPs rapidly change frequencies during the course of sampling, which is most apparent in panels C and G. Panels A–D: trajectories of all SNPs in the populations. Panels E–H: trajectories of only the consensus sequence SNPs identified between the pairings. Panels A and E: B103 longitudinal urine populations. Panels B and F: B103 longitudinal plasma populations. Panels C and G: B103 one week plasma and urine populations. Panels D and H: MS1 longitudinal urine populations. See supplemental Figure 2 for a complete representation of all pairings used in this study.

To better understand the patterns of sequence variability, the HCMV genomic populations were studied as pairs across time or compartments. Eight pairings were created for host-matched specimens, and the frequencies of all SNPs were tracked across the pairings (Figure 2, Figure S2). A complex picture of HCMV evolution emerged from this analysis. For most SNPs, the frequency was either high or low and remained stable between paired specimens (seen as black bands at the top or bottom of the panels). This pattern was observed in all pairings but was most apparent in longitudinal tracking of SNPs in urine populations (Figure 2, Panel A). Indeed, for the 36,131 SNPs tracked for all specimens, 31,919 (88%) changed in frequency by ≤10% (median change = 1.14%). This pattern suggests that the frequencies of the majority of SNPs in these HCMV populations are stable. Figure S2 shows that the populations with the most stable SNPs trajectories (slopes≈0) also appear to have the largest population sizes as measured by viral load (e.g. urine compartment populations) (Table 1). This pattern is consistent with the relationship of drift and population size, in which the effect of drift on the frequencies of neutral SNPs is inversely proportional to population size [12]–[14]. In contrast, subsets of SNPs were observed to rise from low frequency to high frequency or vice versa. This pattern is most apparent when comparing populations sampled across compartments (for example Figure 2, Panel C). Lastly, specimens were collected at 3 time points from patients MS1 and MS2. In these populations, 0.5% of SNPs changed frequency by ≥50% between each time point, moving from low to high to low or vice versa (seen as “V” or inverted “V” patterns in Figure 2, Panel D; Figure S2, Panel H). These patterns suggest that HCMV populations are dynamic and do not completely stabilize in congenitally infected patients during the first year after birth.

**Figure 3:**
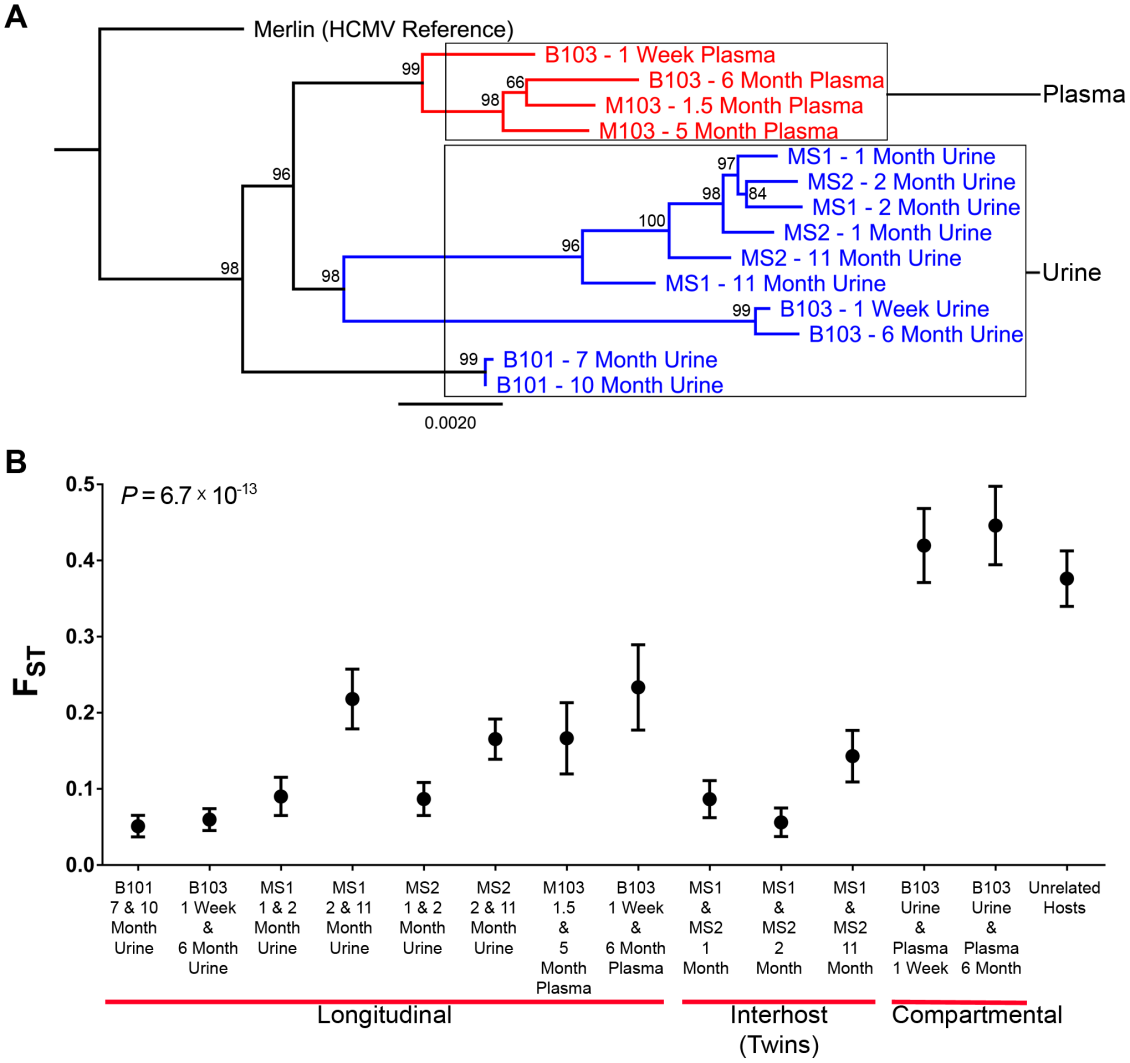
HCMV populations are highly differentiated across compartments. High throughput sequence data was generated on HCMV populations collected from 5 infants from either the urine or plasma compartment or both compartments (patient B103). Panel A: Consensus sequences were generated from the population data of each sample and maximum likelihood phylogenetic trees were constructed from whole genome alignments. Sequences longitudinally sampled from the same compartment of the same host clustered together. In contrast, B103 sequences sampled from the urine and plasma at the same time were highly differentiated and clustered more closely with sequences from other hosts. Intriguingly, plasma sequences from two hosts appeared (M103 and B103) to form a single clade, a result consistent with convergent evolution acting on plasma populations. Node labels represent bootstrap values from 100 replicates and the tree was rooted with the HCMV reference sequence (Strain Merlin, Ref Seq ID: NC_006273). Panel B: Population differentiation between the populations was measured by estimating the summary statistic F_ST_ for the specimen pairings. Higher values of F_ST_ indicate higher levels of population differentiation. HCMV populations are relatively stable across time when measuring populations collected from the same compartment of a single host or between monozygotic, monochorionic twins (MS1 and MS2). B103 populations sampled from different compartments at a single timepoint showed elevated F_ST_ values, indicative of higher levels of population differentiation. The level of differentiation observed between compartments in a single patient is comparable to that observed between populations collected from unrelated patients. Error bars represent 95% confidence intervals. ANOVA analysis: *P* = 6.7×10^−13^.

A consensus sequence was then called for each sampled population. The consensus sequences of two populations of interest were aligned and SNPs identified between the sequences (Table 3 and Figure 2, Panels E–H). Phylogenetic trees were also constructed from all consensus sequences in this study (Figure 3). Longitudinal specimens collected from a single compartment of a single host were closely related as indicated by few SNPs between consensus sequences and short branch lengths on the phylogenetic tree. For example, 49 consensus sequence SNPs were identified between the two B103 urine populations, 20 of these SNPs were non-synonymous. Thus, the consensus sequences differed by only 0.02% at the nucleotide level (π) and 0.04% at the amino acid level (π_AA_) (Table 3). A similar result was observed for longitudinally-sampled plasma populations. For the MS1 and MS2 twins, an average of 149 SNPs (0.08% difference) was identified between urine specimen consensus sequences at the same time point and all MS1 and MS2 sequences clustered together on the phylogenetic tree. In contrast, for infant B103, an order of magnitude increase in the prevalence of consensus sequence SNPs was identified between urine and plasma populations at the same time point. Comparing the consensus sequences of the urine and plasma populations, 1,602 SNPs (∼0.9% difference at the nucleotide and amino acid levels) were identified at 1 week, and 1,771 SNPs (∼1.2% difference) were identified at 6 months. Previously, we have shown that HCMV consensus sequences from different hosts differ by ∼1.1% [6]. Further, the B103 plasma sequences are clearly distant from the B103 urine sequences on the phylogenetic tree (Figure 3). Intriguingly, the urine samples appear as a divergent group on the tree, with B101 sequences being an outlier in this grouping, while the plasma sequences from the two unrelated hosts are closely clustered. This result is consistent with convergent evolution acting on HCMV plasma populations, though the conclusion is tentative given the small sample size. Taken together, these results suggest that HCMV collected from different compartments of a single host are as divergent, or even more divergent, than HCMV collected from unrelated hosts, but that sequences sampled longitudinally from the same compartment of the same host or from monozygotic, monochorionic twins are similar.

**Table 3:** Genome wide longitudinal and compartmental comparisons of HCMV populations.

Comparison	Patient	Sample 1 Source	Sample 1 Time	Sample 2 Source	Sample 2 Time	F_ST_	Consensus Sequence SNPs	Π	Consensus Sequence Nonsynonymous SNPs	Π_AA_
**Longitudinal**	**B101**	Urine	7 month	Urine	10 month	0.05	52	0.03%	10	0.02%
	**B103**	Urine	1 week	Urine	6 months	0.06	49	0.02%	20	0.04%
	**B103**	Plasma	1 week	Plasma	6 months	0.23	213	0.14%	71	0.17%
	**M103**	Plasma	1.5 month	Plasma	5 month	0.16	59	0.06%	33	0.13%
	**MS1**	Urine	1 month	Urine	2 months	0.09	164	0.08%	26	0.05%
	**MS1**	Urine	2 months	Urine	11 months	0.22	234	0.15%	53	0.13%
	**MS2**	Urine	1 month	Urine	2 months	0.08	160	0.07%	52	0.09%
	**MS2**	Urine	2 months	Urine	11 months	0.17	135	0.07%	24	0.04%
**Interhost**	**MS1/MS2**	MS1 Urine	1 month	MS2 Urine	1 month	0.08	147	0.07%	46	0.08%
	**MS1/MS2**	MS1 Urine	2 months	MS2 Urine	2 months	0.05	145	0.07%	26	0.05%
	**MS1/MS2**	MS1 Urine	11 months	MS2 Urine	11 months	0.14	155	0.10%	37	0.09%
**Compartmental**	**B103**	Plasma	1 week	Urine	1 week	0.42	1,602	0.89%	459	0.96%
	**B103**	Plasma	6 months	Urine	6 months	0.45	1,769	1.15%	498	1.21%

In the above analysis, comparisons were made between consensus sequences, which incorporate only high frequency SNPs of the populations. To determine if similar levels of differentiation were present for comparisons of all SNPs, F_ST_ [15], [16] was estimated for the specimen pairings. F_ST_ is a statistic bounded by 0 and 1 that increases with increasing variation in SNP frequencies between populations [17], and is a commonly to measure differentiation between two populations of the same species [18]. In this study, F_ST_ was used to summarize population differentiation either across time or compartments. F_ST_ values of the viral populations in longitudinal populations sampled from urine or plasma compartments were low (average = 0.13, range [0.05–0.23]) (Figure 3, Table 3). Thus, SNP frequencies were generally stable within compartments during the sampling time frame, giving statistical support to the observations from Figure 2. It is noted that the longitudinal F_ST_ values for MS1 and MS2 populations (which were sampled over the longest timespan) rose with the last collection time points (Figure 3). It is unclear whether this trend continued or reached a plateau as no later specimens were available. The highest F_ST_ values, and thus the largest population differentiation, were observed for infant B103 when comparing populations sampled from different compartment specimens (urine and plasma) collected at the same time point (Figure 3, Table 3). F_ST_ was equal to 0.42 at 1 week and 0.45 at 6 months. These values are similar to those estimated between HCMV populations of two unrelated hosts (specifically, urine specimens from infants U01 and U04 that were described previously [6]) – thus, *within host compartment differentiation* can be as great as *between host differentiation* (Figure 3).. These results corroborate the consensus sequence analysis, and suggest that HCMV populations rapidly differentiate between compartments of a single host, but are generally stable over time within the same compartment of a single host or between monozygotic, monochorionic twins.

These data led us to explore the mechanisms of both the population stability and rapid differentiation of the HCMV populations observed in this study. From population genetics, it is known that both demography and selection can lead to large changes in SNP frequencies in relatively short timespans [19]. Demographic events are changes of population size and structure, such as population bottlenecks, expansions and splits, which can lead to stochastic changes in allele frequencies. In contrast, selection is mediated by variations in fitness associated with different alleles and can cause deterministic changes in allele frequency.

**Figure 4:**
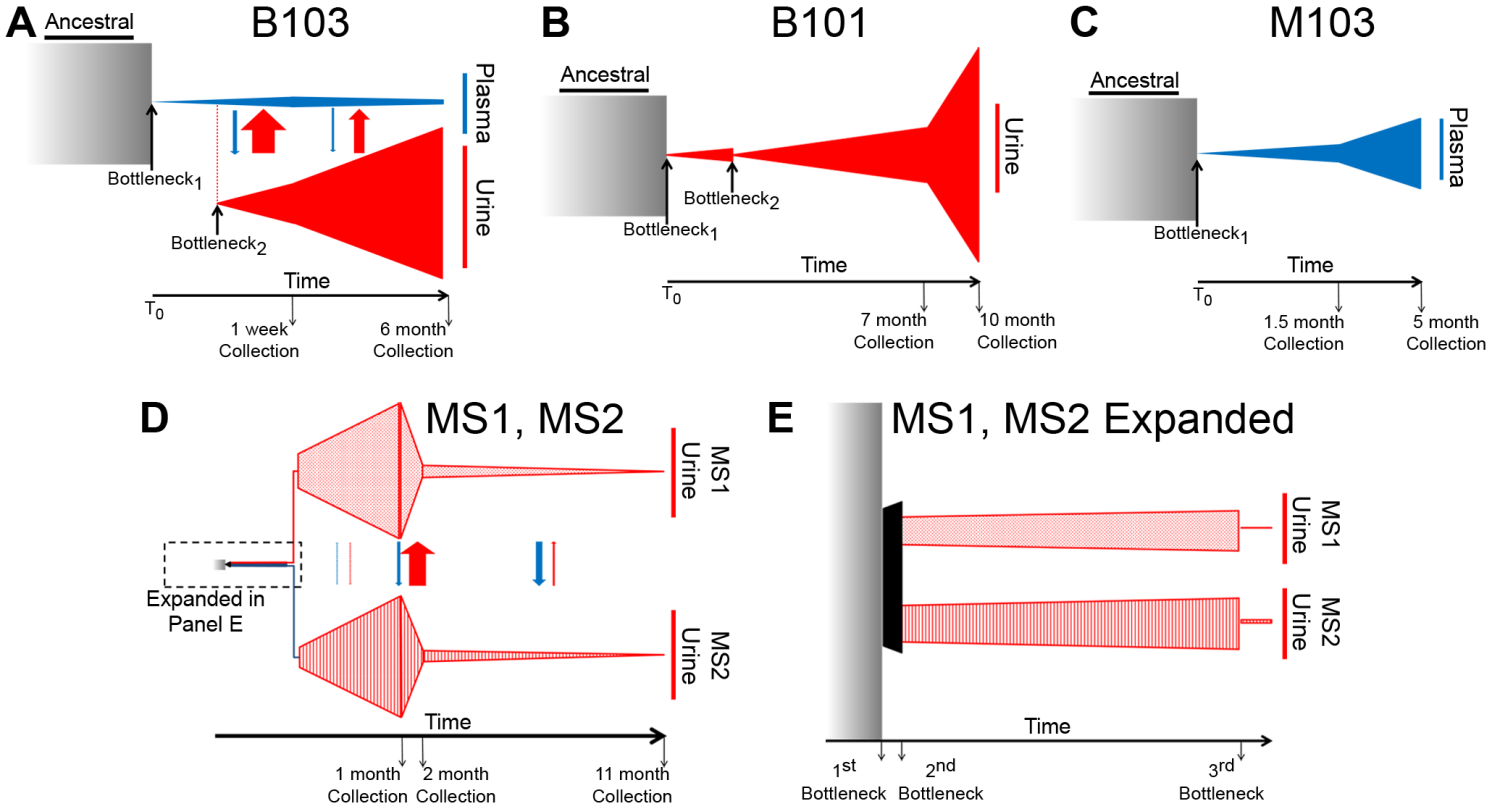
Demographic histories of HCMV clinical isolates show evidence of bottlenecks and expansions. The demographic histories of the viral populations were inferred from the high throughput sequence data. In the models, time increases from left to right and the width of the various shapes is proportional to the size of the viral populations. All population sizes and timespans are drawn to scale. A tabular representation of parameter values of the model can be found in Table S2. See the text for a complete discussion of the models. Panel A: Model of B103 sampled population histories. (The populations within the urine compartment of B103 are drawn to 1∶8 scale [as compared to the B103 plasma compartment] for the sake of clarity.) Panel B: Model of B101 sampled population histories. Panel C: Model of M103 sampled population histories. Panel D: Model of MS1 and MS2 sampled population histories Panel E: Expansion of early timepoints of MS1 and MS2 model. Arrows drawn between populations (Panels A and D) represent migration rates and are scaled relative to each other and not population sizes to improve visibility of the arrows.

The high throughput sequence data was analyzed to infer the demographic history of the viral populations using the statistical framework described previously [20]. This method estimates the parameters of a demographic model by solving the appropriate diffusion equations. This approach has been used to study the demographic history of higher organisms, such as the migration of humans out of Africa [20]. The maximum likelihood values for the parameters of the demographic models were estimated, and uncertainties of the estimates were calculated through non-parametric bootstrapping (Table S2). Using this method, best fit demographic models were developed for the viral populations sampled from the 5 hosts in this study (Figure 4). All population sizes within the model are reported relative to their ancestral population (which is set to 1). In support of modeling accuracy, there was good agreement between the relative population sizes as calculated from the models and as measured empirically with qPCR (Tables 1 and S2).

The demographic history was first inferred for the B103-sampled viral populations given the rapid inter-compartment differentiation observed. In the best-fitting B103 demographic model (Figure 4A), an ancestral population experienced a bottleneck at time 0 and seeded the plasma compartment. The bottleneck reduced population size by approximately 99.7%. The resultant population split 10 weeks later, when 0.41% of the plasma population colonized the urine compartment. The urine population then expanded nearly 2000-fold by the time of the initial collection (1 week postnatally). The urine population continued to expand, growing 4-fold by the second collection at 6 months. In the plasma compartment, the viral population stayed relatively stable throughout the model, increasing in size by approximately 22-fold from the initial bottleneck to the first collection and decreasing by 45% by the 6 months collection. The model also includes asymmetrical migration between the compartments, with 9-fold higher flow of alleles from the urine to the plasma before the first collection and 15-fold higher flow before the second collection (Table S2).

The B101 and M103 best-fit models appear to agree qualitatively with that of B103 with respect to the demographic histories of *in vivo* viral populations (Figures 4B and 4C). B101 and M103 viral populations also experienced bottlenecks at time 0 that reduced population size by 99.1% and 99.8%, respectively (Table S2). For B101, the population experienced a second bottleneck approximately 11 weeks after the initial bottleneck, which reduced population size by 97.6%. The population then expanded and increased in size by ∼1100-fold by the first urine collection (7 months) followed by an additional increase of ∼1.4-fold by the 10 month collection. For M103, the viral population experienced a more modest expansion after the initial (and only detected) bottleneck, increasing in size by ∼140-fold and 2.4-fold by the plasma collections at 1.5 and 5 months after birth, respectively.

Next, a best-fit demographic history was inferred from the MS1 and MS2 sequence data (Figures 4D–E, Table S2). This model differed from the previous models in several substantial ways. First, the initial bottleneck did not occur at time 0, but rather at 0.466 months after time 0 (i.e. the earliest event inferred in the model). Second, the previous models of populations sampled from the urine compartments (B101 and B103) contained 2 bottlenecks in the population histories, while the best-fit model of MS1 and MS2 contained 3 bottlenecks, with the first bottleneck shared between the twins. Of note, the first 2 bottlenecks of the MS1 and MS2 model occurred sequentially, with contiguous confidence intervals for the timing of the bottlenecks (Table S2). In addition, these bottlenecks were less severe than those from other models, but the net effect of the 2 sequential bottlenecks was to reduce population size by 95% and 92% in the MS1 and MS2 lineages, respectively. Together, these reductions in population size are comparable to those inferred in the other models for a single bottleneck. Possible causes for the disagreements between the models are relayed in the Discussion.

From the demographic model, some or all of the differentiation across compartments may be explained by a bottleneck effect associated with plasma populations colonizing the urine compartment, such as shown in Figure 4A. However, positive selection may also contribute to rapid differentiation due to the fixation of advantageous alleles as the virus moves to a new host environment. To test for evidence of positive selection within HCMV populations, the population branch statistic (PBS) [21] was employed. PBS measures localized increases in population differentiation between two closely related populations and a third outlier population [21], with larger PBS values indicative of a higher probability that positive selection has locally altered SNP frequencies. The test has high power to detect recent positive selection, particularly when the selected SNP rises from standing variation [21], an important consideration for this work. An HCMV population sampled from an unrelated host (described previously [6]) was used as the outlier in this study, and simulations under the demographic models in Figure 4 were used to determine the 5% significance threshold (Figure S5).

**Figure 5:**
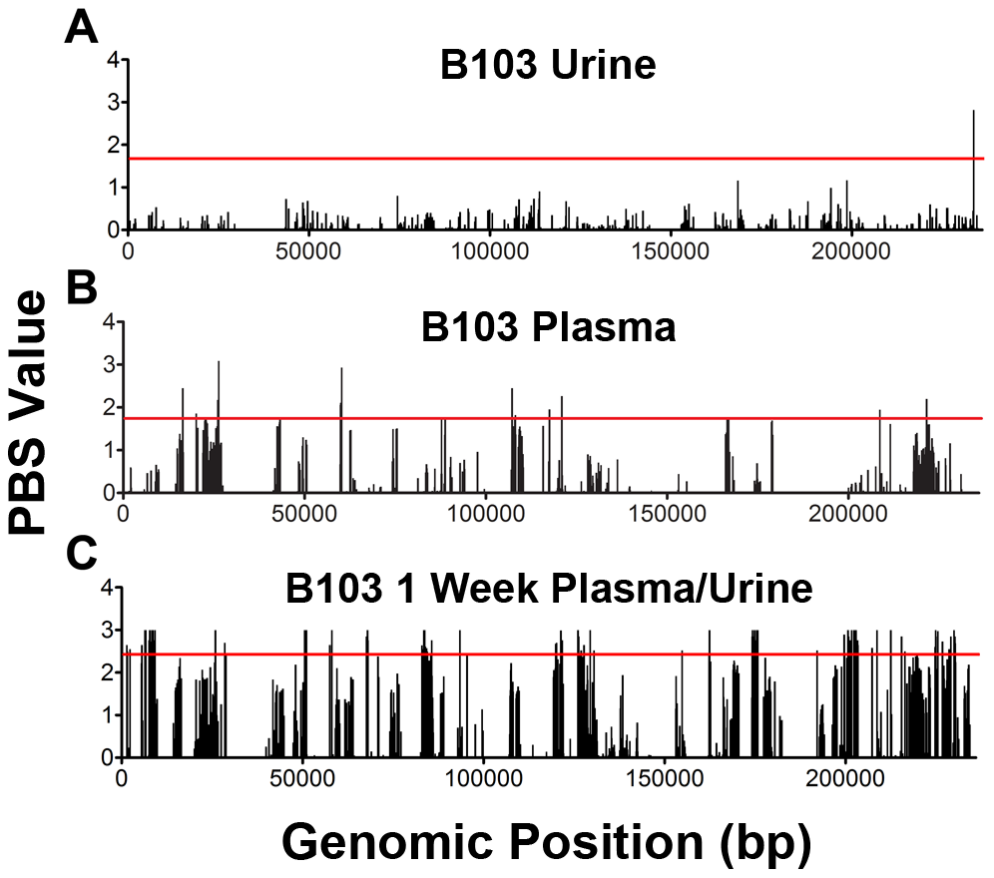
Positive selection plays a variable role in HCMV in vivo evolution. The population branch statistic (PBS) was used to identify putative targets of positive selection within the HCMV viral populations collected from congenitally infected infants. Higher PBS values identify loci that have a higher likelihood of being targets of postive selection. The red line indicates the 5% significance threshold, above which values are considered significant. This threshold was determined through simulations using the demographic parameters inferred from the data, as depicted in Figure 4 and Table S2. See Tables S4, S5, S6, S7, S8, S9, S10, S11, S12, S13, S14 for tabular presentations of these data. Panel A: longitudinal B103 urine populations. Panel B: B103 longitudinal plasma populations. Panel C: B103 1 week plasma and urine populations. For the results of all PBS analyses, see Figure S3.

The results of this analysis show that the effect of positive selection in shaping HCMV evolution is variable and dependent on context. Very few putative targets of positive selection were identified in the compartment-matched, longitudinal specimens from all infants (Figures 5 and S3, Tables 4 and S2, S3, S4, S5). In these populations, an average of 10 total SNPs and 3 non-synonymous SNPs were identified in the screen. *UL7*, *UL13* and *UL73* (encoding gN) were identified as putative targets of positive selection in several populations, suggesting that variation in these genes can affect viral fitness among different hosts. Additionally, few targets were identified when screening for beneficial SNPs associated with the urine compartments of monozygotic twins (MS1 and MS2) (Figure S3, Table 4 and S6, S7, S8, S9, S10, 11), where on an average 12 total SNPs and 2 non-synonymous SNPs were identified in each host at the 3 collection time points.

**Table 4:** Summary of putative targets of positive selection identified in HCMV populations.

Comparison	Patient	Source - Sample 1	Time - Sample 1	Source - Sample 2	Time - Sample 2	Positively Selected SNPs	Selected SNPs in Coding Region^1^	ORFs^2^ with a Selected SNP^3^	Nonsynonymous Selected SNPs^4^
Longitudinal	B101	Urine	7 month	Urine	10 month	11	8	4	3
	B103	Urine	1 week	Urine	6 month	2	2	1	2
	B103	Plasma	1 week	Plasma	6 month	23	20	13	8
	M103	Plasma	1.5 month	Plasma	5 month	3	3	3	0
Interhost	MS1/MS2	MS1 Urine	1 month	MS2 Urine	1 month	23	21	6	2
	MS1/MS2	MS1 Urine	2 months	MS2 Urine	2 months	13	11	4	2
	MS1/MS2	MS1 Urine	11 months	MS2 Urine	11 months	9	8	3	2
	MS1/MS2	MS2 Urine	1 month	MS1 Urine	1 month	9	7	3	1
	MS1/MS2	MS2 Urine	2 months	MS1 Urine	2 months	11	11	3	7
	MS1/MS2	MS2 Urine	11 months	MS1 Urine	11 months	6	6	2	0
Compartmental	B103	Plasma	1 week	Urine	1 week	114	83	34	26

1 The number of positively selected SNPs located in a coding region.

2 Open Reading Frame.

3 The number of ORFs that contain a positively selected SNP.

4 The number of positively selected SNPs that are nonsynonymous.

In contrast, the assay yielded significantly different results when studying positive selection associated with movement of virus across compartments. The B103 populations sampled from the 1 week urine and plasma specimens were used to estimate the effect of positive selection associated with colonization of a new host compartment. 114 SNPs detected in the B103 urine compartment were identified as putative targets of positive selection, including 31 in non-coding regions and 83 in coding regions (Figure 5, Tables 4 and S13). The coding SNPs were located in a total of 34 ORFs. Comparison of screens of positive selection (Table 4) showed that the urine compartment colonization result was a significant outlier (Grubbs test, *P* = 6.8×10^−4^). These data suggest that positive selection is an important contributor to the rapid evolution of HCMV populations associated with colonization of distinct tissue compartments.

Many of the SNPs detected in the screen of selection associated with urine compartment colonization showed positional clustering (Figure S4), suggesting that only a few SNPs were positively selected and the remainder were swept to high frequency via hitchhiking [22], [23]. To identify which SNPs associated with compartment colonization may be targets of selection and not simply linked neutral variants, the coding SNPs were classified as synonymous or non-synonymous (the presumed targets of positive selection). Of the 83 coding SNPs, 26 were non-synonymous mutations located in 16 ORFs (Tables 4 and S12). Within this group, several targets have been shown to exhibit strain or inter-host variability, such as *RL5A* [24] and *UL55* (encoding gB) [25]. Thus, these data extend previous studies of HCMV polymorphisms by showing that differentiation can exist within hosts as well as between hosts, and that differentiation can be driven by positive selection. Because selection was observed in the urine compartment (i.e., kidney), these results suggest that the *RL5A* and *UL55* SNPs offer a fitness advantage in this compartment, possibly due to their effects on cell tropism as suggested previously [26]. Positively selected nonsynonymous SNPs were also localized to *US2*, *US3*, and *US7*, which are contained within the *US2* through *US11* block of ORFs that are important for immune evasion by interference of the antigen presentation machinery [27]. Many ORFs of unknown function were identified in this screen, some with multiple nonsynonymous SNPs, such as *UL124*. However, the functional effects of these identified SNPs are still unknown.

**Figure 6:**
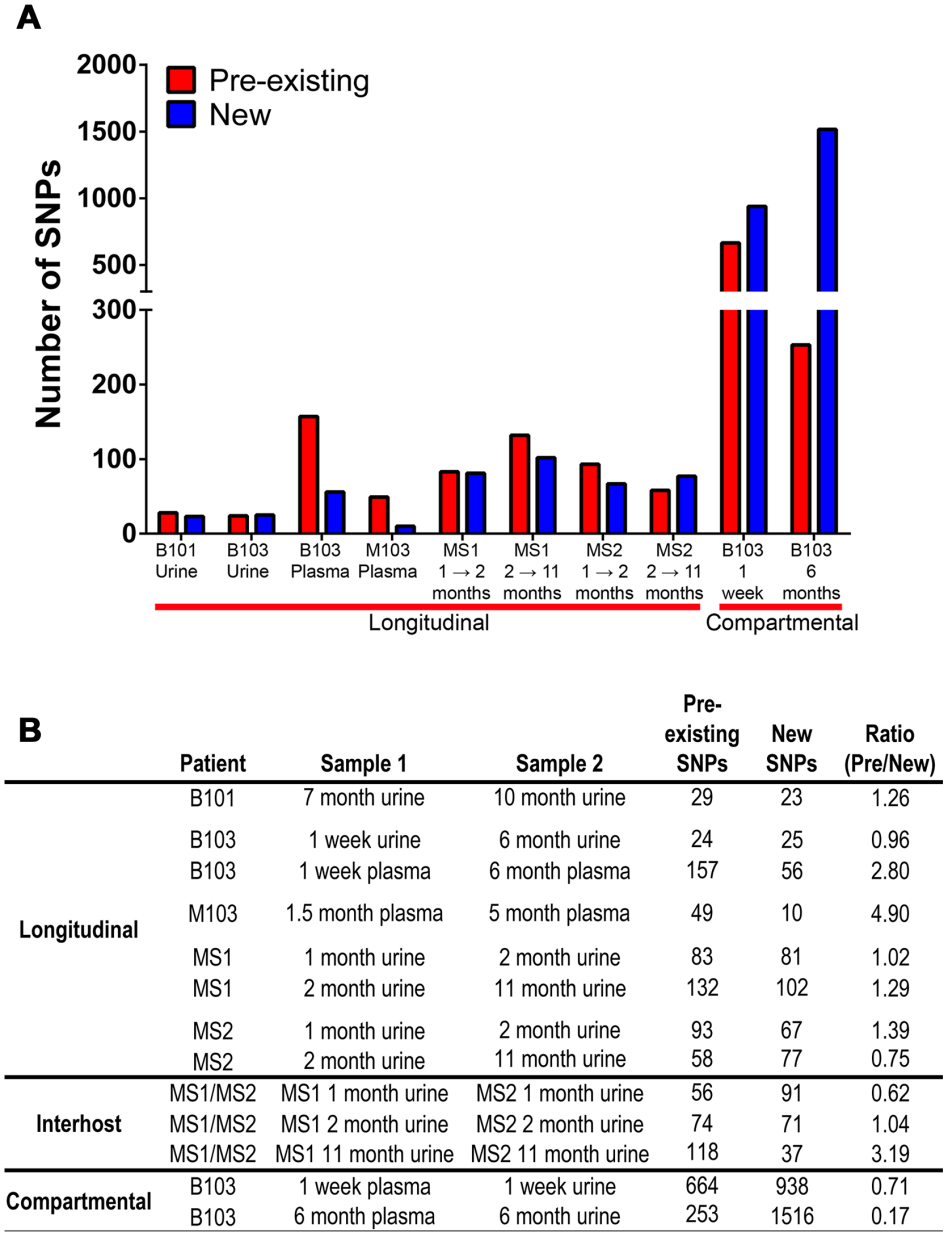
Pre-existing and new SNPs contribute to varying degrees to HCMV evolution. Panel A: Consensus sequence SNPs between populations were classified as either pre-existing (detected in the ancestral and derived populations) or new (only detected in the derived population). Plotted are the occurrence of these two classes of SNPs in the various patient specimens. Panel B: A tabular representation of the data presented in Panel A.

Lastly, it was observed that positively selected SNPs (Tables S4, S5, S6, S7, S8, S9, S10, S11, S12, S13, S14) were both pre-existing (i.e., identified in the ancestral population) and newly arising (i.e., not identified in the ancestral population). The relative contribution of each in driving differentiation was calculated using genome wide data. The analysis showed that both pre-existing and novel SNPs contribute to HCMV evolution (Figure 6), though there was a significant difference between the relative contributions of the two classes (Chi-square, *P* = 1.4×10^−11^). Specifically, pre-existing SNPs were more frequently identified in longitudinal plasma populations, while novel SNPs were more prevalent in the cross-compartment comparisons. In total, these results suggest that both pre-existing and *de novo* mutations contribute with varying degrees to the evolution of HCMV within human hosts.

## Discussion

HCMV has been shown to exhibit significant inter-host sequence divergence as well as high levels of intra-host variability. Here we provide a detailed study of the evolution of HCMV in human hosts using specimens collected across tissue compartments and time. Furthermore, we provide evidence of rapid evolution of HCMV populations associated with movement of the virus between host compartments. The rapid divergence can best be explained by a demographic history that includes population bottlenecks and expansions, as well as positive selection across a subset of loci.

This study shows that the evolution of HCMV within human hosts can proceed in two ways. HCMV populations are relatively stable within tissue compartments. For example, the urine populations of B101 and B103 exhibited only 52 and 49 consensus sequence SNPs, respectively, and low F_ST_ values. This result is in agreement with previous studies that have monitored HCMV genetic sequences within patients [5], [28]. In addition, HCMV populations were stable across hosts, albeit for the unique case of monozygotic, monochorionic twins when sampled from the same compartment. In these cases, demographic modeling suggested stable population size and structure, and the trajectory of many SNPs is consistent with the effects of drift.

In contrast, HCMV populations can also rapidly evolve during colonization of different compartments within a host. Significant HCMV inter-host differentiation is well documented, both at the genetic and genomic levels [3], [6], [24], [29], [30]. Here we show that a similar level of differentiation can be observed within different compartments of a single host (here represented by the plasma and urine). However, HCMV can be found in many other organs [4] and bodily fluids (e.g., saliva, breast milk, and semen). While the large differentiation between compartments and the patterns of bottlenecks and expansions observed in our study may be unique to plasma and urine, any movement between other compartments would likely involve similar demographic histories. Moreover, each compartment may have unique selective pressures as reported here for the plasma and urine compartments. It is therefore probable that most compartments will show levels of differentiation similar to those reported in this study.

One reason that the plasma and urine compartments were chosen for study is because they represent the circulating and shed HCMV populations, respectively. Plasma virus is likely limited to the individual (with the important exceptions of transfusion/transplantation-associated and congenital infections) whereas virus shed into urine is excreted and possibly infecting other hosts. The results in this work suggest that there are large differences between viral populations isolated from these two sources. The potential implications of these findings are numerous. For example, do studies of secreted virus, such as from the urine and/or saliva, provide useful information about circulating virus or infection-associated diseases? In addition, how do SNPs associated with shedding viral populations (e.g., urine populations) affect tropism for mucosal surfaces, the presumed route of most inter-host infections? The evidence for positive selection when crossing into the urine compartment suggests that the viral population has adapted for increased fitness, but most likely increased fitness in renal epithelial cells. Thus, it is not known how compartmentalization affects inter-host infectivity. Alternatively, because at least a portion of the differences between populations could be explained by the neutral demographic history, some of the SNPs would have arisen stochastically and may have little or no effect on viral fitness. Therefore, it will be interesting to determine the phenotypic differences between patient-derived HCMV populations from different compartments.

Demographic histories of HCMV populations were generated from population data in this study. These demographic models may provide a partial explanation for the population differentiation observed across compartments. The B103 model suggests that the urine and plasma populations split 3 months prior to the initial collection. It was also observed that these populations are highly differentiated, as measured by F_ST_ (Figure 2, Table 3). From the B103 demographic model, at least a part of this differentiation may be explained by the strong bottleneck and large population expansion that occurs as the virus colonized the urine compartment. The bottleneck would reduce population size, leading to decreased diversity due to the stochastic elimination of ancestral SNPs (which likely explains the results shown in Figure 1). A decreased population size would also increase the speed and probability of fixation of neutral SNPs [13], [14]. Secondly, DNA replication associated with population expansion could introduce new mutations to the urine compartment population.

The demographic model generated from B103 data also suggests a novel mechanism for reinfection. In the model, there was asymmetric migration of alleles between host compartments with a higher flow from the urine to the plasma. In this study, the flow of alleles was relatively low and the plasma population size was fairly stable. One could speculate that after a collapse of the plasma population, due the effects of the adaptive immune response or antiviral therapy, migration between compartments would provide a source of highly differentiated virus. Thus, a clinical manifestation of this phenomenon could be intrahost (i.e. self) reinfection by virus sourced from various compartments. If this is true, effective HCMV vaccine design will need to account for not only plasma-associated epitopes but also epitopes sourced from other compartments. Future studies are needed to test the validity and prevalence of this mechanism of reinfection, and its potential effect on anti-HCMV clinical strategies.

An important disagreement was noted between the B101 and B103 models and those of MS1 and MS2: the best-fit model of B101 and B103 urine populations contained 2 bottlenecks separated by ∼2.5 months, while the MS1 and MS2 model contained 3 bottlenecks, separated by 0.2 months and 2.2 months. One possible explanation for the disagreement is that in some hosts, HCMV undergoes 2 bottlenecks en route to the urine compartment, while in other hosts there are 3 bottlenecks. However, there is no clear biological mechanism to explain this difference. Alternatively, the MS1 and MS2 models appear to infer events further back in time than those of the other models. Thus, the first bottleneck in this model could reflect an earlier event for which the other datasets had insufficient power to detect. A third explanation is that the MS1 and MS2 models have higher-resolution than those inferred from the other datasets. To generate these models, one compares the spectrum of neutral allele frequencies of the experimental dataset to the spectra that would be expected from populations that have experienced various demographic events (i.e. bottlenecks and/or expansions). The best fit model is one for which the experimental spectrum most closely matches the expected spectrum. Unfortunately, models of various population histories could generate similar allele frequency spectra, limiting the ability to fully resolve these models. However, because MS1 and MS2 viral populations were derived from the same ancestral population, two allele frequency spectra were estimated from populations that evolved in parallel from the same ancestor, providing more information about the evolutionary history of the populations and allowing similar models to be resolved. Thus, it is proposed that all HCMV urine populations have experienced at least 3 bottlenecks prior to colonization of the kidney. However, two bottlenecks may not be resolved by data from a single host due to the very close timing of the bottlenecks (∼1 week). The net effect of both bottlenecks is reduction of population size by >90% (Table S2).

If three bottlenecks do indeed occur during the course of HCMV congenital infections, the biological cause of these reductions in population size is not known. We hypothesize that the first bottleneck results from the movement of the virus from the maternal compartment to the placenta. In all models, the timing of this event was ∼13–18 weeks gestational age and appears to agree with the known epidemiology and pathology of symptomatic HCMV congenital infections [31]–[33]. In the MS1 and MS2 models, the first bottleneck is shared and followed by a ∼1 week period in which the viral population expanded ∼4-fold before splitting and entering the separate, second bottlenecks of the two infant lineages. We propose that these events reflect replication of the virus in the twin's shared placenta, followed by independent infection of the fetal circulatory compartments. Replication of HCMV in placental cytotrophoblasts has been demonstrated experimentally [34], and the data presented here offer a novel method to support the hypothesis that HCMV replicates within the placental compartment. We further hypothesize that the third bottleneck in the history of the urine populations represents movement of the virus from the plasma to the renal compartment, which occurred ∼9–11 weeks after the initial fetal infection event. Whether the timing and extent of the bottlenecks is consistent across HCMV congenital infections or differs between symptomatic and asymptomatic infections still needs to be studied. Nevertheless, our work demonstrates that viral demographic histories can be clinically informative for understanding viral infections.

From the data in this study, it is tempting to speculate on the relative contribution of demography and selection to the evolution of HCMV populations. For example, 1602 SNPs were observed between the B103 urine and plasma compartment consensus sequences at 1 week of age (Table 3), of which 114 were putative targets of positive selection (Table 4). One could propose that ∼7% of SNPs rose in frequency due to positive selection (either directly or via linkage), and the remainder were governed by genetic drift. However, caution must be used with these estimates. For example, a demographic model was first fit to the data, and then the signatures of positive selection were detected, which may result in over-fitting the demographic model [35]. Second, the test of positive selection may have excluded SNPs because of missing data due to incomplete coverage of the outlier group. Third, the time course of selection may overlap poorly with the time course of sample collection. For example, a SNP may have arisen from very low frequency to become fixed due to strong selection. A changing selective environment may have reduced or eliminated the selective advantage, allowing the SNP to drift to a lower frequency in the population. If the timing of sampling does not overlap the timing of selection, the SNP would not be identified as a target of positive selection. Thus, we are limited in our ability to accurately quantitate the relative contribution of demography and selection to the evolutionary patterns observed in this study. However, it is very reasonable to state based on the data as a whole that both demography and positive selection influence the intrahost evolution of HCMV, and thus both need to be accounted for in future studies of the natural history of HCMV and other viral infections.

## Materials and Methods

### Ethics statement

The use of specimens from subjects B101, B103, and M103 was approved by the University of Massachusetts Medical School and Baystate Medical Center Institutional Review Boards. Subjects MS1 and MS2 clinical specimens were obtained from neonates with congenital HCMV infection during the course of routine clinical care at the University of Minnesota Medical School. Protocols for collection of HCMV isolates from congenitally infected infants were approved by the University of Minnesota Institutional Review Board. Informed consent was obtained from subjects' parents for study of HCMV.

### Patient population, collection of specimens and viral DNA extraction

Patients identified at the University of Minnesota Medical Center or University of Massachusetts Memorial Health Center were evaluated for HCMV infection on the basis of signs and symptoms suggesting congenital infection. Patients MS1 and MS2 were monochorionic, monozygotic twins with clinical evidence of congenital CMV consisting of thrombocytopenia, transaminitis, and, for MS1, a small gestational age phenotype. Congenital infection was confirmed for all patients by urine HCMV positive cultures before 3 weeks of age and/or PCR detection of HCMV DNA. No patients were treated with antiviral drugs.

Serial specimens were collected at times described in Table 1. Specimens were stored at −80°C until DNA purification. Total DNA was purified using a Qiagen Blood and Tissue Kit using the standard protocol.

### Amplification of HCMV DNA

A set of primer pairs were constructed that spanned the entire HCMV genome. Details of the primers sequences and primer design strategy have been described [6]. The conditions for PCR were as follows: 1× PfuUltra II PCR buffer, 0.25 mM each dNTP (NEB), 0.25 uM each primer (IDT DNA), 0.5 uL PfuUltra II Polymerase (Agilent) and 1M betaine. A touchdown PCR was run on an Eppendorf Mastercycler ep gradient S with the following program for all reactions: 98°C for 2 min, 5 cycles of 98°C for 30 s, 63°C (decreasing by 1°/cycle) for 30 s, 72°C for 2 min, followed by 25 cycles of 98°C for 30 s, 58°C for 30 s and 72°C for 2 min, with a 10 min final extension at 72°C. Approximately 10,000 genomes copies, based on qPCR results, were used for each amplification reaction. All amplified products were size-selected on agarose gels and gel purified. After amplification of the HCMV genome, all amplicons were quantified on a Nanodrop 2000, pooled in equimolar proportions and used as substrate in Illumina sequencing. Quantitative PCR was performed using primers and probes described previously [36].

### Illumina sequencing

The DNA in pooled amplicons was sheared by sonication on a Sonic Dismembrator 550 (Fisher) until the median size was ∼350 basepairs (bp). The DNA library was prepared as described [37]. Briefly, DNA was end-repaired using the End-Repair Enzyme Mix (NEB), and A-tailed using the ATP and Klenow (exo^−^) (NEB). Adapters with appropriate barcodes were ligated onto the modified DNA ends. The library was then size selected on a 2% agarose gel, to produce a library with a median size of 350 bp+/−50 bp. The library was amplified with Illumina primers (P/N 1003454) (www.illumina.com). Once prepared, the libraries were combined in appropriate ratios and submitted for paired-end sequencing on the Illumina GAII. A Toledo strain amplicon set generated from a BAC clone was included as an internal control for measuring error rates.

### Sequence analysis

The raw sequence images were processed through Illumina Pipeline 1.8 to generate sequence data. The consensus sequence of each sample was called as described [6]. Variants from the consensus sequence were called using the variant filtering protocol as described previously [6]. The variants were filtered to remove errors using parameter thresholds. Variants with parameters below the thresholds were discarded. The thresholds were: minimum basecall quality (≥33), mapping quality (≥89), and depth (≥15). Variant frequency was also used as a threshold. In our prior work, a variant frequency threshold of ≥1.9% was used. However, due to reduced error rates associated with Illumina sequencing, a lower variant frequency threshold of ≥1.02% could be used to achieve a 5% false positive rate (Figure S1). Unfolded SNP frequency spectra were generated using the HCMV reference sequence (Strain Merlin, Ref Seq ID: NC_006273) as an outgroup for the intrahost populations under study.

Whole genome alignments were generated using the Vista whole genome aligner hosted on the Vista server [38]. Maximum likelihood trees were constructed using PhyML software [39] with an HKY85 substitution model, transition/transversion rates estimated from the data, and 100 bootstrap replicates.

### Modeling of demographic history of HCMV populations

A dataset of only synonymous mutations for each viral population was created. These datasets were used as neutral datasets for subsequent analysis of demographic history and were analyzed with the program *dadi* (Diffusion Approximation for Demographic Inference) [20] to build demographic models and yield maximum likelihood estimates of the parameters of the models. SNP frequency data from the pooled sequencing data was projected down into 15 bins, which is the minimum sequencing depth required for SNP calling. For the M103 and B101 populations, samples were collected at two time points from a single host and a single compartment. The models built from these data used the “frozen” function of *dadi*, in which the model was constructed with the joint frequency spectrum from both time points and the early time point sample was “frozen” (no input of additional mutations and drift ceased) after the collection time point. Samples collected from B103 were collected at multiple time points and two compartments, and samples from MS1 and MS2 were collected at 3 time points and two hosts. In these cases, the models depicted in Figure 4 reflect the best-fit model that was constructed from the two divergent populations at the final time point. Specifically, the models were built from the 6 month urine and plasma samples from B103 and the 11 month urine samples from MS1 and MS2. However, models were also built using the earlier time point data from different compartments or hosts (for example, 1 week urine and plasma from B103) (data not shown). The earlier time point data were used to build a framework of the model, which eased the building of the later, more complex model. Many models were tested, including a standard neutral model and models excluding migration or alternating the number of bottlenecks. The alternate models were found to be worse fits to the experimental data than the models depicted in Figure 4, as measured by log likelihoods and AIC model comparisons. A fit of the models are depicted in Figure S5. 95% confidence intervals of the parameters were estimated using nonparametric bootstrapping. In the resulting models, all times are reported in units of 2N generations. However, the time between the collection time points was known (e.g., 1 week to 6 months of age). Timespans were used to convert all times within the models to calendar time. Migration rates in the model are given in units of M = 2N_e_m, where N_e_ is the effective population size and m is the number of migrants per generation. N_e_ was estimated from the variance in SNP frequency in time sampled populations using the method of Jorde and Ryman [40]. The estimate of N_e_ for all samples was approximately 1000 (mean = 961, range [478–1450]) (Table S3) and this value was used to convert parameters from the model to units of migrants per generation.

### Test of positive selection

To test for evidence of positive selection, we employed the population branch statistic (PBS) as described [21]. The PBS statistic uses a log transformation of the F_ST_ statistic to identify regions of the genome that have an elevated level of differentiation between closely related populations. Positive selection acting on an allele would be predicted to locally increase F_ST_ at the site of the selection, and thus, lead to an increase in the PBS statistic in this region. Importantly, F_ST_-based tests show high power to detect selection from populations with high levels of standing variation [41], [42]. This was an important characteristic for a test of selection because we have previously shown that HCMV populations collected from congenitally infected neonates are highly variable. We also employed a test of selection that searched for localized regions of reduced variability and an excess of surrounding high frequency alleles [43]. This pattern is expected when selection has recently acted upon a new mutation from a population with low mutation rates. This test identified fewer targets of selection within these data, but there was high concordance between this test and the PBS statistic (data not shown).

To determine the significance of the PBS statistic, 10,000 simulations were run for a 5,000 bp region under the inferred demographic histories of the populations (Figure 4) using the forward simulator *sfs_code* [44]. Simulation of a full length HCMV genome (∼235,000 bp) was computationally intensive and required a prohibitively long time to complete. For this reason, sequence length in the simulation was set to be similar to an average HCMV gene with surrounding regulatory elements, following a previously described approach [21]. The 10,000 iterations were analyzed as a complete dataset, and each position contained at least 1 SNP. The SNP at each position (5,000 in total) with the maximum PBS value was identified from the simulations (Figure S6). The results from the simulations were then compared to the experimental data to determine the 5% significance threshold of the PBS values and the corresponding *P* values of all SNPs with significant (*P*<0.05) PBS scores.

### Availability of data

Raw sequencing reads from Illumina sequencing will be deposited in the Sequence Read Archive (http://www.ncbi.nlm.nih.gov/Traces/sra/sra.cgi). Data will be available upon publication.

## Supporting Information

Figure S1 SNP calling false positive rate (FPR) is dependent on filtering threshold. A filtering algorithm that has been described previously [6] was employed to reduce false positives when calling SNPs from high throughput sequence data. A parameter of the filtering is SNP frequency threshold, such that SNPs with frequencies below the threshold are excluded. Here is plotted the relationship between the FPR and the frequency threshold values used in filtering. These values were calculated by sequencing a BAC clone, which was assumed to contain no polymorphisms and all errors were assumed to be amplification or sequencing errors.This relationship was plotted for three average sequencing depths (650×, 1500× and 3000×). The dotted black line represents a 5% FPR.
(TIF)

Figure S2 HCMV populations show patterns of both stability and differentiation. Full panel of results from those depicted in figure 2. Plotted here are the trajectories of SNPs between sample pairings. Panels A–H: trajectories of all SNPs in the populations. Panels I–P: trajectories of only consensus SNPs identified between the pairings. Panels A, I: B101 longitudinal urine samples. Panels B, J: B103 longitudinal urine samples. Panels C, K: M103 longitudinal plasma samples. Panels D, L: B103 longitudinal plasma samples. Panels E, M: B103 1 week urine and plasma samples. Panels F, N: B103 6 month urine and plasma samples. Panels G, O: MS1 longitudinal urine samples. Panels H, P: MS2 longitudinal urine samples.
(TIF)

Figure S3 Evidence of positive selection in HCMV samples. Full results of PBS tests from Figure 5. The population branch statistic (PBS), a measure of the signature of positive selection, was plotted across the HCMV genome. Higher values are indicative of a higher probability of a region being the target of the positive selection. Red dotted line represents the 5% significance threshold. Panel A: longitudinal B101 urine samples. Panel B: longitudinal B103 urine samples. Panel C: longitudinal M103 plasma samples. Panel D: longitudinal B103 plasma samples. Panel E: B103 1 week urine and plasma samples. Panel F: MS1 1 month urine sample. Panel G: MS1 2 month urine sample. Panel H: MS1 11 month urine sample. Panel I: MS2 1 month urine sample. Panel J: MS2 2 month urine sample. Panel K: MS2 11 month urine sample.
(TIF)

Figure S4 Targets of positive selection are clustered on the HCMV genome. SNPs from the B103 urine sample were identified as putative targets of positive selection. Plotted as a histogram are distances between putatively selected SNPs. The majority of putatively selected SNPs are located within 200 basepairs of the nearest selected SNP.
(TIF)

Figure S5 Comparison of demographic models to population data. Joint allele frequency spectra of the population data (top left of each panel) are compared to the expected joint allele spectra of the demographic models depicted in Figure 4 (top right of each panel). Residuals between the population data and demographic models are plotted as frequency spectra (bottom left of each panel) or as histograms (bottom right of each panel). Log likelihood of the models are shown at the top of each panel. Panel A: B103 Panel B: B101 Panel C: M103 Panel D: MS1 and MS2.
(TIF)

Figure S6 Results of simulations under demographic models depicted in Figure 4. 10,000 simulations were run for a 5,000 basepair region under the inferred demographic histories of the populations (Figure 3) using the forward simulator *sfs_code* [44]. The SNP at each position (5,000 in total) with the maximum PBS value was identified from the simulations. The 5% significance thresholds for each simulation set are shown as red lines. Panel A: longitudinal B101 urine samples. Panel B: longitudinal B103 urine samples. Panel C: longitudinal M103 plasma samples. Panel D: longitudinal B103 plasma samples. Panel E: B103 1 week urine and plasma samples. Panel F: MS1 1 month urine sample. Panel G: MS1 2 month urine sample. Panel H: MS1 11 month urine sample. Panel I: MS2 1 month urine sample. Panel J: MS2 2 month urine sample. Panel K: MS2 11 month urine sample.
(TIF)

Table S1 Summary of sequencing data.
(PDF)

Table S2 Parameter values of demographic models HCMV populations.
(PDF)

Table S3 Estimate of HCMV effective population size from time sampled populations.
(PDF)

Table S4 Targets of positive selection in 6 month B103 plasma populations.
(PDF)

Table S5 Targets of positive selection in 6 month B103 urine populations.
(PDF)

Table S6 Targets of positive selection in 10 month B101 urine populations.
(PDF)

Table S7 Targets of positive selection in 5 month M103 plasma populations.
(PDF)

Table S8 Targets of positive selection in MS1 1 month urine populations.
(PDF)

Table S9 Targets of positive selection in MS2 1 month urine populations.
(PDF)

Table S10 Targets of positive selection in MS1 2 month urine populations.
(PDF)

Table S11 Targets of positive selection in MS2 2 month urine populations.
(PDF)

Table S12 Targets of positive selection in MS1 11 month urine populations.
(PDF)

Table S13 Targets of positive selection in MS2 11 month urine populations.
(PDF)

Table S14 Targets of positive selection in 1 week B103 urine populations.
(PDF)

## References

[1] Dowd JB, Aiello AE, Alley DE (2009) Socioeconomic disparities in the seroprevalence of cytomegalovirus infection in the US population: NHANES III. Epidemiol Infect 137: 58–65. 18413004 10.1017/S0950268808000551 PMC3806637

[2] Cannon MJ (2009) Congenital cytomegalovirus (CMV) epidemiology and awareness. J Clin Virol 46 Suppl 4: S6–10. 19800841 10.1016/j.jcv.2009.09.002

[3] McGeoch DJ, Rixon FJ, Davison AJ (2006) Topics in herpesvirus genomics and evolution. Virus Res 117: 90–104. 16490275 10.1016/j.virusres.2006.01.002

[4] Bhattacharjee B, Renzette N, Kowalik TF (2012) Genetic Analysis of Cytomegalovirus in Malignant Gliomas. Journal of Virology 86: 6815–6824. 22496213 10.1128/JVI.00015-12 PMC3393585

[5] Görzer I, Guelly C, Trajanoski S, Puchhammer-Stockl E (2010) Deep sequencing reveals highly complex dynamics of human cytomegalovirus genotypes in transplant patients over time. J Virol JVI.00475-00410. 10.1128/JVI.00475-10 PMC2898262 20463084

[6] Renzette N, Bhattacharjee B, Jensen JD, Gibson L, Kowalik TF (2011) Extensive Genome-Wide Variability of Human Cytomegalovirus in Congenitally Infected Infants. PLoS Pathog 7: e1001344. 21625576 10.1371/journal.ppat.1001344 PMC3098220

[7] Davison AJ (2011) Evolution of sexually transmitted and sexually transmissible human herpesviruses. Annals of the New York Academy of Sciences 1230: E37–E49. 22417106 10.1111/j.1749-6632.2011.06358.x

[8] McGeoch DJ (1989) The Genomes of the Human Herpesviruses: Contents, Relationships, and Evolution. Annual Review of Microbiology 43: 235–265. 10.1146/annurev.mi.43.100189.001315 2552898

[9] Davison AJ, Dolan A, Akter P, Addison C, Dargan DJ, et al (2003) The human cytomegalovirus genome revisited: comparison with the chimpanzee cytomegalovirus genome. J Gen Virol 84: 17–28. 12533697 10.1099/vir.0.18606-0

[10] Dolan A, Cunningham C, Hector RD, Hassan-Walker AF, Lee L, et al (2004) Genetic content of wild-type human cytomegalovirus. J Gen Virol 85: 1301–1312. 15105547 10.1099/vir.0.79888-0

[11] Mocarski ES (2002) Immunomodulation by cytomegaloviruses: manipulative strategies beyond evasion. Trends in Microbiology 10: 332–339. 12110212 10.1016/s0966-842x(02)02393-4

[12] Kimura M (1983) The Neutral Theory of Molecular Evolution. Cambridge: Cambridge University Press.

[13] Kimura M (1962) On the probability of fixation of mutant genes in a population. Genetics 47: 713–719. 14456043 10.1093/genetics/47.6.713 PMC1210364

[14] Kimura M, Ohta T (1969) The Average Number of Generations until Fixation of a Mutant Gene in a Finite Population. Genetics 61: 763–771. 17248440 10.1093/genetics/61.3.763 PMC1212239

[15] Weir BS, Cockerham CC (1984) Estimating F-Statistics for the Analysis of Population Structure. Evolution 38: 1358–1370. 10.1111/j.1558-5646.1984.tb05657.x 28563791

[16] Wright S (1949) The Genetical Structure of Populations. Annals of Human Genetics 15: 323–354. 10.1111/j.1469-1809.1949.tb02451.x 24540312

[17] Holsinger KE, Weir BS (2009) Genetics in geographically structured populations: defining, estimating and interpreting FST. Nat Rev Genet 10: 639–650. 19687804 10.1038/nrg2611 PMC4687486

[18] Weir BS, Hill WG (2002) Estimating F-statistics. Annu Rev Genet 36: 721–750. 12359738 10.1146/annurev.genet.36.050802.093940

[19] Thornton KR, Jensen JD, Becquet C, Andolfatto P (2007) Progress and prospects in mapping recent selection in the genome. Heredity 98: 340–348. 17473869 10.1038/sj.hdy.6800967

[20] Gutenkunst RN, Hernandez RD, Williamson SH, Bustamante CD (2009) Inferring the joint demographic history of multiple populations from multidimensional SNP frequency data. PLoS Genet 5: e1000695. 19851460 10.1371/journal.pgen.1000695 PMC2760211

[21] Yi X, Liang Y, Huerta-Sanchez E, Jin X, Cuo ZX, et al (2010) Sequencing of 50 human exomes reveals adaptation to high altitude. Science 329: 75–78. 20595611 10.1126/science.1190371 PMC3711608

[22] Kaplan NL, Hudson RR, Langley CH (1989) The “hitchhiking effect” revisited. Genetics 123: 887–899. 2612899 10.1093/genetics/123.4.887 PMC1203897

[23] Maynard-Smith J, Haigh J (1974) The hitch-hiking effect of a favourable gene. Genet Res 23: 23–35. 4407212

[24] Bradley AJ, Lurain NS, Ghazal P, Trivedi U, Cunningham C, et al (2009) High-throughput sequence analysis of variants of human cytomegalovirus strains Towne and AD169. J Gen Virol 90: 2375–2380. 19553388 10.1099/vir.0.013250-0 PMC2885757

[25] Chou S (1992) Comparative analysis of sequence variation in gp116 and gp55 components of glycoprotein B of human cytomegalovirus. Virology 188: 388–390. 1314465 10.1016/0042-6822(92)90771-g

[26] Meyer-König U, Vogelberg C, Bongarts A, Kampa D, Delbrück R, et al (1998) Glycoprotein B genotype correlates with cell tropism in vivo of human cytomegalovirus infection. Journal of Medical Virology 55: 75–81. 9580889

[27] Tortorella D, Gewurz BE, Furman MH, Schust DJ, Ploegh HL (2000) Viral Subversion of the Immune System. Annual Review of Immunology 18: 861–926. 10.1146/annurev.immunol.18.1.861 10837078

[28] Stanton R, Westmoreland D, Fox JD, Davison AJ, Wilkinson GW (2005) Stability of human cytomegalovirus genotypes in persistently infected renal transplant recipients. J Med Virol 75: 42–46. 15543586 10.1002/jmv.20235

[29] Bradley AJ, Kovács IJ, Gatherer D, Dargan DJ, Alkharsah KR, et al (2008) Genotypic analysis of two hypervariable human cytomegalovirus genes. J Med Virol 80: 1615–1623. 18649324 10.1002/jmv.21241 PMC2658010

[30] Cunningham C, Gatherer D, Hilfrich B, Baluchova K, Dargan DJ, et al (2009) Sequences of complete human cytomegalovirus genomes from infected cell cultures and clinical specimens. J Gen Virol 91: 605–615. 19906940 10.1099/vir.0.015891-0 PMC2885759

[31] Stagno S PRFCG, et al (1986) Primary cytomegalovirus infection in pregnancy: Incidence, transmission to fetus, and clinical outcome. JAMA: The Journal of the American Medical Association 256: 1904–1908. 3020264

[32] Pereira L, Maidji E, McDonagh S, Genbacev O, Fisher S (2003) Human cytomegalovirus transmission from the uterus to the placenta correlates with the presence of pathogenic bacteria and maternal immunity. J Virol 77: 13301–13314. 14645586 10.1128/JVI.77.24.13301-13314.2003 PMC296088

[33] Lazzarotto T, Gabrielli L, Foschini MP, Lanari M, Guerra B, et al (2003) Congenital cytomegalovirus infection in twin pregnancies: viral load in the amniotic fluid and pregnancy outcome. Pediatrics 112: e153–157. 12897321 10.1542/peds.112.2.e153

[34] Fisher S, Genbacev O, Maidji E, Pereira L (2000) Human Cytomegalovirus Infection of Placental Cytotrophoblasts In Vitro and In Utero: Implications for Transmission and Pathogenesis. Journal of Virology 74: 6808–6820. 10888620 10.1128/jvi.74.15.6808-6820.2000 PMC112198

[35] Crisci JL, Poh YP, Bean A, Simkin A, Jensen JD (2012) Recent progress in polymorphism-based population genetic inference. J Hered 103: 287–296. 22246406 10.1093/jhered/esr128

[36] Gault E, Michel Y, Dehee A, Belabani C, Nicolas JC, et al (2001) Quantification of human cytomegalovirus DNA by real-time PCR. J Clin Microbiol 39: 772–775. 11158149 10.1128/JCM.39.2.772-775.2001 PMC87818

[37] Quail MA, Kozarewa I, Smith F, Scally A, Stephens PJ, et al (2008) A large genome center's improvements to the Illumina sequencing system. Nat Meth 5: 1005–1010. 10.1038/nmeth.1270 PMC2610436 19034268

[38] Frazer KA, Pachter L, Poliakov A, Rubin EM, Dubchak I (2004) VISTA: computational tools for comparative genomics. Nucleic Acids Res 32: W273–279. 15215394 10.1093/nar/gkh458 PMC441596

[39] Guindon S, Gascuel O (2003) A simple, fast, and accurate algorithm to estimate large phylogenies by maximum likelihood. Syst Biol 52: 696–704. 14530136 10.1080/10635150390235520

[40] Jorde PE, Ryman N (2007) Unbiased estimator for genetic drift and effective population size. Genetics 177: 927–935. 17720927 10.1534/genetics.107.075481 PMC2034655

[41] Innan H, Kim Y (2008) Detecting local adaptation using the joint sampling of polymorphism data in the parental and derived populations. Genetics 179: 1713–1720. 18562650 10.1534/genetics.108.086835 PMC2475763

[42] Hermisson J, Pennings PS (2005) Soft sweeps: molecular population genetics of adaptation from standing genetic variation. Genetics 169: 2335–2352. 15716498 10.1534/genetics.104.036947 PMC1449620

[43] Nielsen R, Williamson S, Kim Y, Hubisz MJ, Clark AG, et al (2005) Genomic scans for selective sweeps using SNP data. Genome Res 15: 1566–1575. 16251466 10.1101/gr.4252305 PMC1310644

[44] Hernandez RD (2008) A flexible forward simulator for populations subject to selection and demography. Bioinformatics 24: 2786–2787. 18842601 10.1093/bioinformatics/btn522 PMC2639268

[45] Nei M, Li WH (1979) Mathematical model for studying genetic variation in terms of restriction endonucleases. Proc Natl Acad Sci U S A 76: 5269–5273. 291943 10.1073/pnas.76.10.5269 PMC413122

