# Supplementary figures and images for: Rapid Intrahost Evolution of Human Cytomegalovirus Is Shaped by Demography and Positive Selection
Source: PLoS Genet. 2013 Sep 26;9(9):e1003735. doi: 10.1371/journal.pgen.1003735 (PMC3784496; doi:10.1371/journal.pgen.1003735)

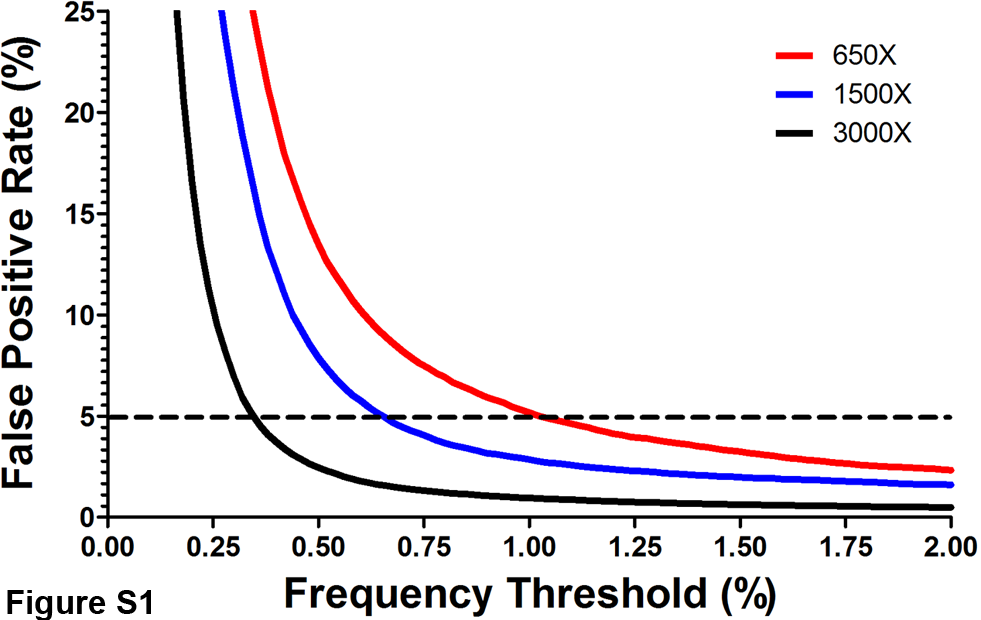

Supplement: Figure S1 — SNP calling false positive rate (FPR) is dependent on filtering threshold. A filtering algorithm that has been described previously [6] was employed to reduce false positives when calling SNPs from high throughput sequence data. A parameter of the filtering is SNP frequency threshold, such that SNPs with frequencies below the threshold are excluded. Here is plotted the relationship between the FPR and the frequency threshold values used in filtering. These values were calculated by sequencing a BAC clone, which was assumed to contain no polymorphisms and all errors were assumed to be amplification or sequencing errors.This relationship was plotted for three average sequencing depths (650×, 1500× and 3000×). The dotted black line represents a 5% FPR. (TIF) [file pgen.1003735.s001.tif]

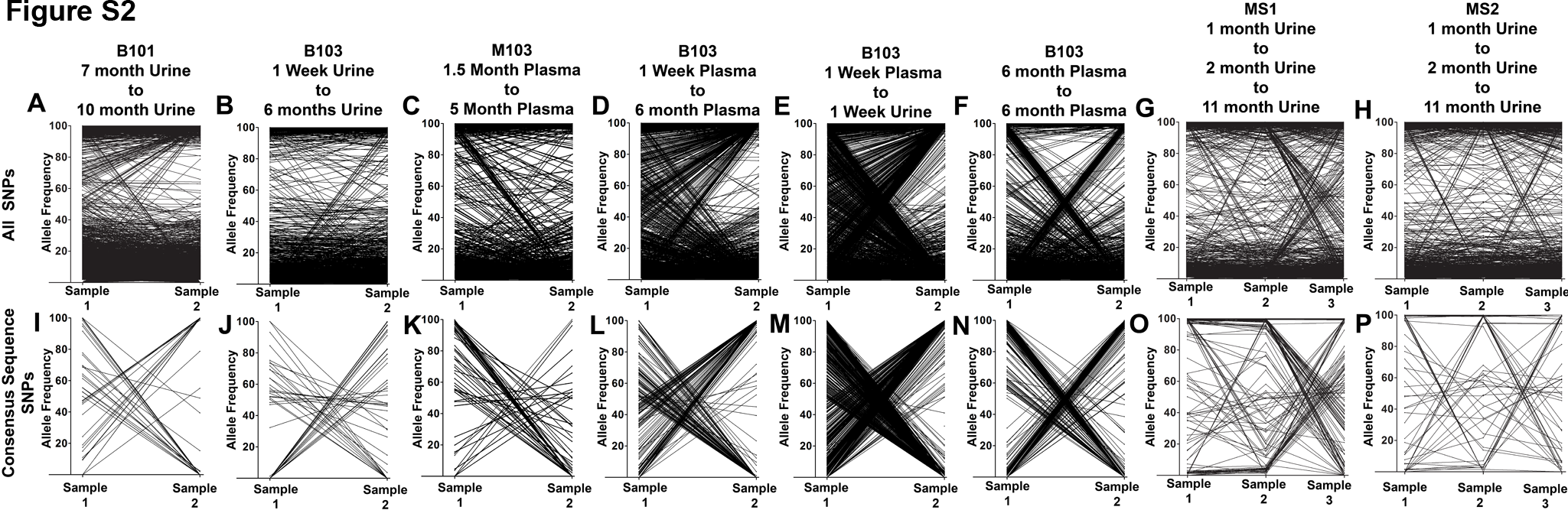

Supplement: Figure S2 — HCMV populations show patterns of both stability and differentiation. Full panel of results from those depicted in figure 2. Plotted here are the trajectories of SNPs between sample pairings. Panels A–H: trajectories of all SNPs in the populations. Panels I–P: trajectories of only consensus SNPs identified between the pairings. Panels A, I: B101 longitudinal urine samples. Panels B, J: B103 longitudinal urine samples. Panels C, K: M103 longitudinal plasma samples. Panels D, L: B103 longitudinal plasma samples. Panels E, M: B103 1 week urine and plasma samples. Panels F, N: B103 6 month urine and plasma samples. Panels G, O: MS1 longitudinal urine samples. Panels H, P: MS2 longitudinal urine samples. (TIF) [file pgen.1003735.s002.tif]

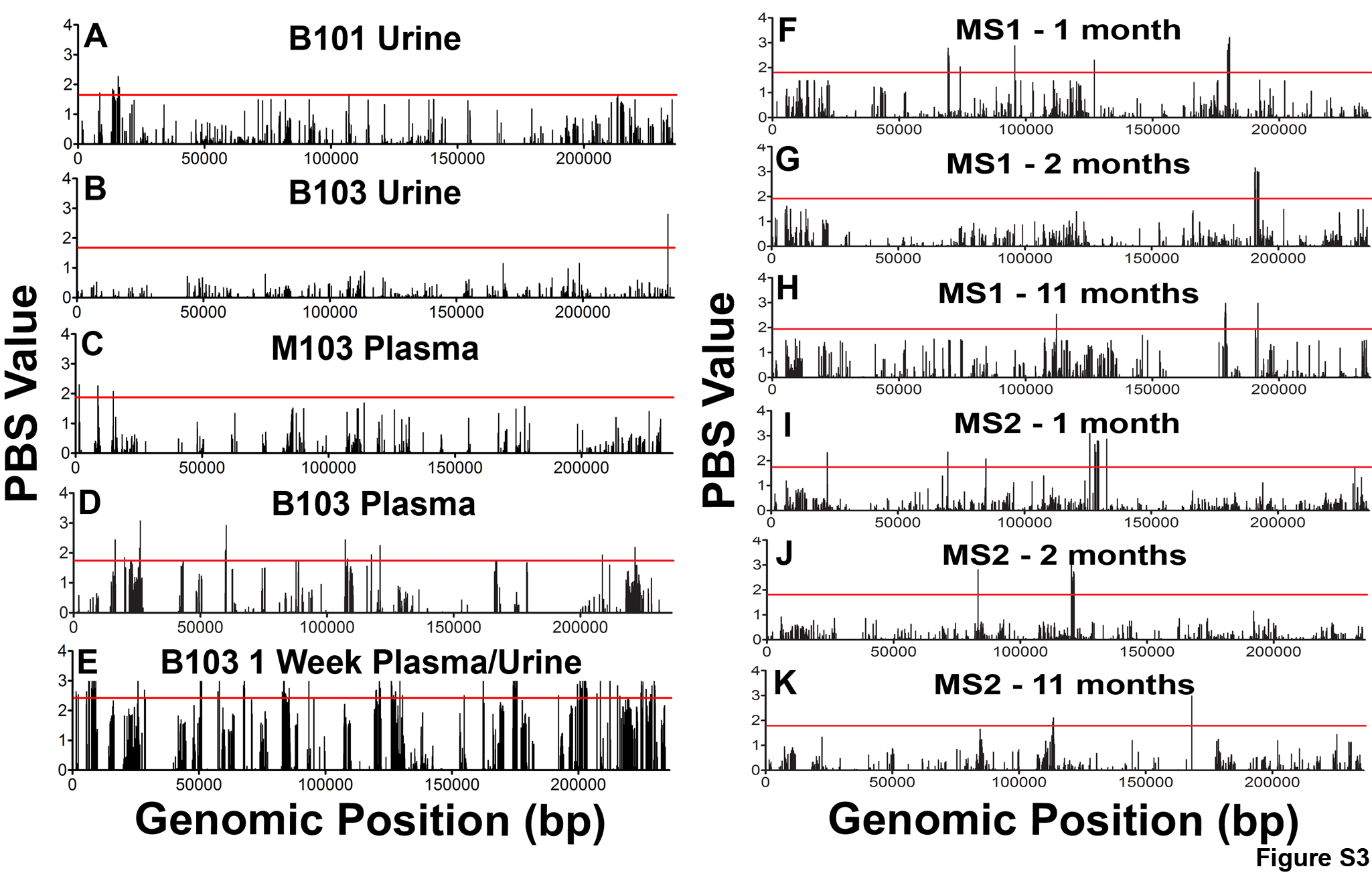

Supplement: Figure S3 — Evidence of positive selection in HCMV samples. Full results of PBS tests from Figure 5. The population branch statistic (PBS), a measure of the signature of positive selection, was plotted across the HCMV genome. Higher values are indicative of a higher probability of a region being the target of the positive selection. Red dotted line represents the 5% significance threshold. Panel A: longitudinal B101 urine samples. Panel B: longitudinal B103 urine samples. Panel C: longitudinal M103 plasma samples. Panel D: longitudinal B103 plasma samples. Panel E: B103 1 week urine and plasma samples. Panel F: MS1 1 month urine sample. Panel G: MS1 2 month urine sample. Panel H: MS1 11 month urine sample. Panel I: MS2 1 month urine sample. Panel J: MS2 2 month urine sample. Panel K: MS2 11 month urine sample. (TIF) [file pgen.1003735.s003.tif]

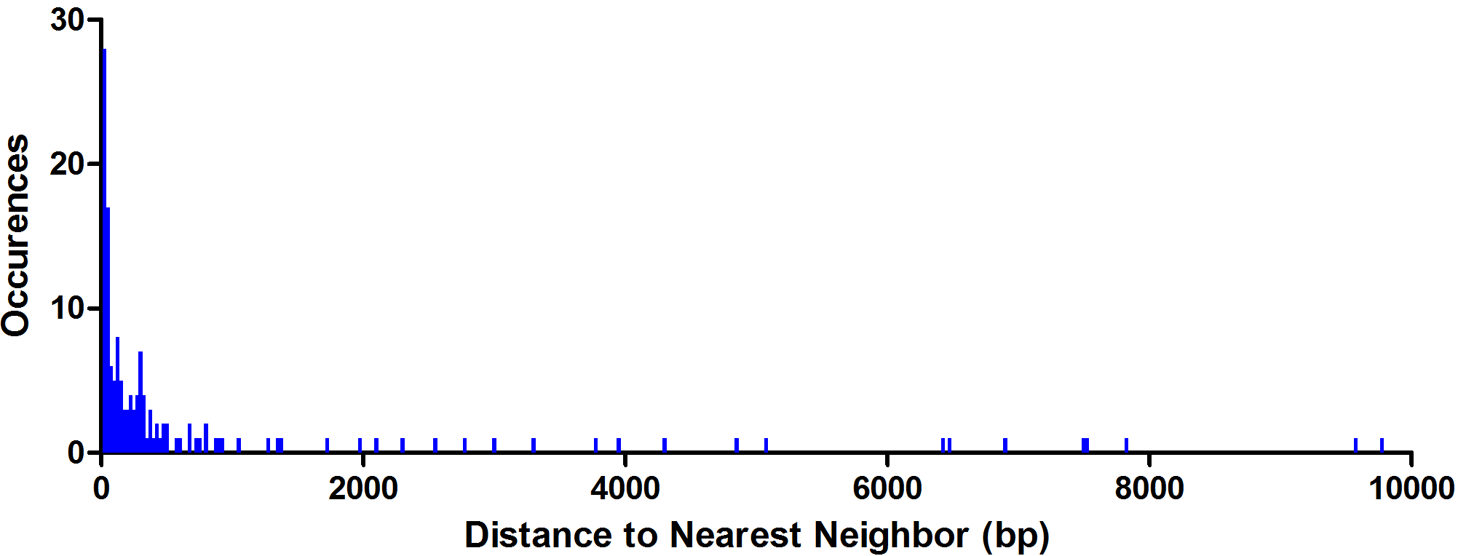

Supplement: Figure S4 — Targets of positive selection are clustered on the HCMV genome. SNPs from the B103 urine sample were identified as putative targets of positive selection. Plotted as a histogram are distances between putatively selected SNPs. The majority of putatively selected SNPs are located within 200 basepairs of the nearest selected SNP. (TIF) [file pgen.1003735.s004.tif]

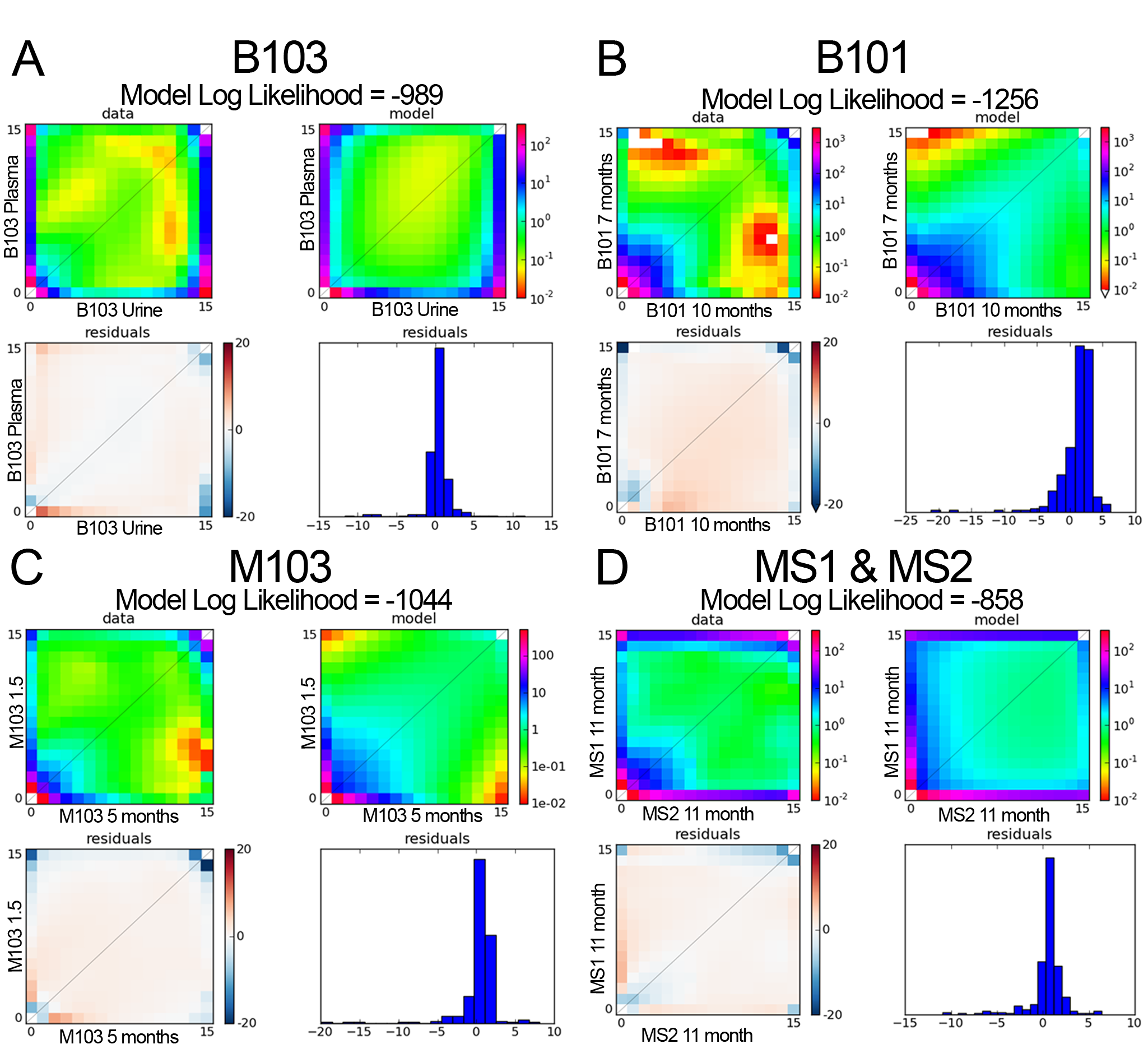

Supplement: Figure S5 — Comparison of demographic models to population data. Joint allele frequency spectra of the population data (top left of each panel) are compared to the expected joint allele spectra of the demographic models depicted in Figure 4 (top right of each panel). Residuals between the population data and demographic models are plotted as frequency spectra (bottom left of each panel) or as histograms (bottom right of each panel). Log likelihood of the models are shown at the top of each panel. Panel A: B103 Panel B: B101 Panel C: M103 Panel D: MS1 and MS2. (TIF) [file pgen.1003735.s005.tif]

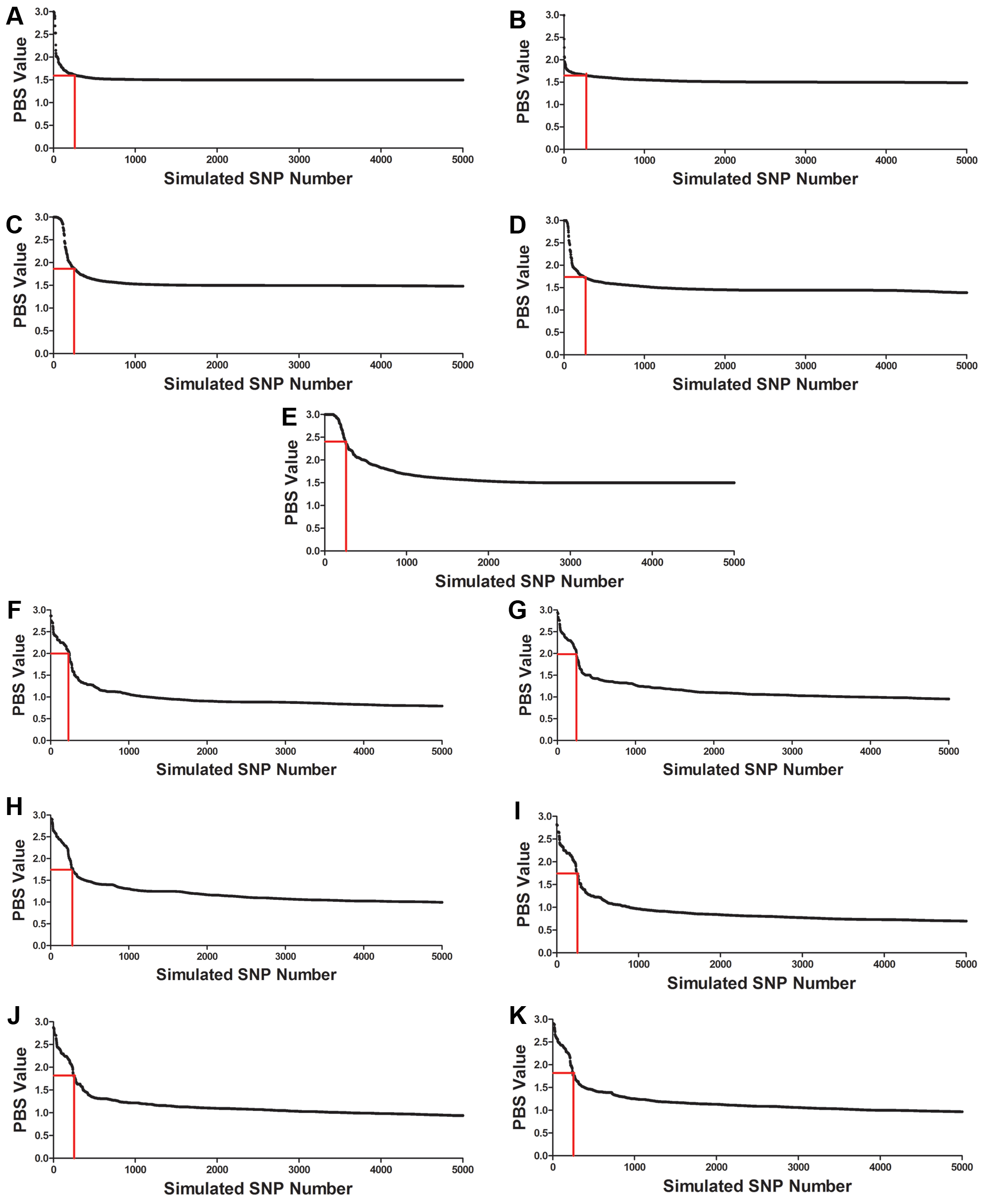

Supplement: Figure S6 — Results of simulations under demographic models depicted in Figure 4. 10,000 simulations were run for a 5,000 basepair region under the inferred demographic histories of the populations (Figure 3) using the forward simulator sfs_code [44]. The SNP at each position (5,000 in total) with the maximum PBS value was identified from the simulations. The 5% significance thresholds for each simulation set are shown as red lines. Panel A: longitudinal B101 urine samples. Panel B: longitudinal B103 urine samples. Panel C: longitudinal M103 plasma samples. Panel D: longitudinal B103 plasma samples. Panel E: B103 1 week urine and plasma samples. Panel F: MS1 1 month urine sample. Panel G: MS1 2 month urine sample. Panel H: MS1 11 month urine sample. Panel I: MS2 1 month urine sample. Panel J: MS2 2 month urine sample. Panel K: MS2 11 month urine sample. (TIF) [file pgen.1003735.s006.tif]
