# Supplementary material for: Rapid Intrahost Evolution of Human Cytomegalovirus Is Shaped by Demography and Positive Selection
Source: PLoS Genet. 2013 Sep 26;9(9):e1003735. doi: 10.1371/journal.pgen.1003735 (PMC3784496; doi:10.1371/journal.pgen.1003735)
Supplement: Table S1 — Summary of sequencing data. (PDF) [file pgen.1003735.s007.pdf]

Table S1: Summary of Sequencing Data

Patient	Sample Type	Time of Collection	Total Reads	Mapped Reads	Mapped Output (Mb)	Depth	Coverage
B101	Urine	7 months	9.53E+06	4.6E+06	1.5E+08	641	94.7%
B101	Urine	10 months	1.50E+07	6.0E+06	2.0E+08	845	89.9%
B103	Urine	1 week	1.67E+07	1.2E+07	4.1E+08	1725	88.6%
B103	Urine	6 months	2.54E+07	2.2E+07	7.3E+08	3101	89.7%
B103	Plasma	1 week	2.5E+07	9.9E+06	3.3E+08	1383	76.2%
B103	Plasma	6 months	2.2E+07	1.3E+07	4.4E+08	1862	66.0%
M103	Plasma	1.5 months	2.14E+07	8.1E+06	2.7E+08	1135	63.3%
M103	Plasma	5 months	1.89E+07	7.1E+06	2.3E+08	994	55.4%
MS1	Urine	1 month	3.20E+07	1.98E+07	6.6E+08	2781	97.6%
MS1	Urine	2 months	2.17E+07	1.24E+07	4.1E+08	1737	94.1%
MS1	Urine	11 months	3.87E+07	1.55E+07	5.1E+08	2176	88.1%
MS2	Urine	1 month	2.99E+07	1.69E+07	5.5E+08	2346	96.3%
MS2	Urine	2 months	3.89E+07	3.33E+07	1.1E+09	4663	95.5%
MS2	Urine	11 months	1.71E+07	1.08E+07	3.5E+08	1506	94.7%
