# Supplementary material for: Rapid Intrahost Evolution of Human Cytomegalovirus Is Shaped by Demography and Positive Selection
Source: PLoS Genet. 2013 Sep 26;9(9):e1003735. doi: 10.1371/journal.pgen.1003735 (PMC3784496; doi:10.1371/journal.pgen.1003735)
Supplement: Table S2 — Parameter values of demographic models HCMV populations. (PDF) [file pgen.1003735.s008.pdf]

Table S2: Parameter Values of Demographic Models of HCMV Populations

B103		
	**Time (months)**	**95% Confidence Interval**
$T_{Bottleneck1}$	0	---
$T_{Bottleneck2\text{-}Split}$	2.33	1.98 - 2.69
$T_{Collection1}$	5.33	4.34 - 6.33
$T_{Collection2}$	11.1	10.1 - 12.1
	**Relative Population Size**	**95% Confidence Interval**
$N_{Ancestral}$	1	---
$N_{Collection1\text{-}Plasma}$	0.0732	.0582 - .0883
$N_{Collection2\text{-}Plasma}$	0.0410	.0305 - .0516
$N_{Collection1\text{-}Urine}$	2.60	1.91 - 3.28
$N_{Collection2\text{-}Urine}$	9.92	7.90 - 11.9
$Bottleneck_{1st}$	0.33%	.26% - .39%
$Bottleneck_{2nd}$	0.41%	.31% - .50%
	**Migration Rate (x 10^{-5} migrants per generation)**	**95% Confidence Interval**
$m_{Urine\text{-}to\text{-}Plasma1}$	137	108 - 167
$m_{Plasma\text{-}to\text{-}Urine1}$	15.1	10.5 - 19.8
$m_{Urine\text{-}to\text{-}Plasma2}$	57.4	44.2 - 70.5
$m_{Plasma\text{-}to\text{-}Urine2}$	3.90	3.31 - 4.55

B101		
	**Time (months)**	**95% Confidence Interval**
$T_{Bottleneck1}$	0	---
$T_{Bottleneck2}$	2.54	1.90 - 3.18
$T_{Collection1}$	11.1	10.4 - 11.7
$T_{Collection2}$	14.1	13.7 - 14.4

	Relative Population Size	95% Confidence Interval
$N_{Ancestral}$	1	---
$N_{Pre\text{-}Bottleneck2}$	0.172	.138 - .206
$N_{Collection1}$	4.31	3.30 - 5.32
$N_{Collection2}$	6.18	4.82 - 7.53
$Bottleneck_{1st}$	0.87%	.75% - 1.00%
$Bottleneck_{2nd}$	2.36%	1.97% - 2.74%

M103

	Time (months)	95% Confidence Interval
$T_{bottleneck}$	0	---
$T_{Collection1}$	6.24	5.70 - 6.80
$T_{Collection2}$	9.75	9.58 - 9.91
	**Relative Population Size**	**95% Confidence Interval**
$N_{Ancestral}$	1	---
$N_{Collection1}$	0.283	.226 - .341
$N_{Collection2}$	0.669	.584 - .754
Bottleneck	0.20%	.15% - .26%

MS1 & MS2

	Time (Months)	95% Confidence Interval
$T_{Bottleneck1}$	0.466	.333 - .600
$T_{Bottleneck2\text{-}Split}$	0.623	.600 - .646
$T_{Bottleneck3}$	2.86	2.81 - 2.92
$T_{Collection1}$	6.62	6.14 - 7.10
$T_{Collection2}$	7.49	7.38 - 7.60
$T_{Collection3}$	16.6	15.7 - 17.5
	**Relative Population Size**	**95% Confidence Interval**
$N_{Ancestral}$	1	---

$N_{Pre\text{-}Bottleneck2}$	0.413	.351 - .475
$N_{Pre\text{-}Bottleneck3\text{-}MS1}$	0.105	.083 - .127
$N_{Pre\text{-}Bottleneck3\text{-}MS2}$	0.142	.120 - .165
$N_{Collection1\text{-}MS1}$	13.2	11.4 - 15.1
$N_{Collection1\text{-}MS2}$	11.2	9.4 - 13.1
$N_{Collection2\text{-}MS1}$	1.37	1.18 - 1.57
$N_{Collection2\text{-}MS2}$	1.16	1.02 - 1.30
$N_{Collection3\text{-}MS1}$	0.139	.124 - .154
$N_{Collection3\text{-}MS2}$	0.117	.085 - .149
**$Bottleneck_1$**	10.6%	8.55% - 12.7%
**$Bottleneck_{2\text{-}MS1}$**	11.7%	7.9% - 15.5%
**$Bottleneck_{2\text{-}MS2}$**	20.0%	14.1% - 26.0%
**$Bottleneck_{3\text{-}MS1}$**	1.51%	1.16% - 1.86%
**$Bottleneck_{3\text{-}MS2}$**	6.10%	4.94% - 7.26%
	**Migration Rate (x 10^{-5} migrants per generation)**	**95% Confidence Interval**
$m_{MS1\text{-}to\text{-}MS2\text{-}1}$	3.32	2.93 - 3.71
$m_{MS2\text{-}to\text{-}MS1\text{-}1}$	1.76	1.12 - 2.41
$m_{MS1\text{-}to\text{-}MS2\text{-}2}$	92.7	83.4 - 102
$m_{MS2\text{-}to\text{-}MS1\text{-}2}$	1112	921 - 1304
$m_{MS1\text{-}to\text{-}MS2\text{-}3}$	371	322 - 419
$m_{MS2\text{-}to\text{-}MS1\text{-}3}$	21.3	12.4 - 30.3
