# Supplementary material for: Rapid Intrahost Evolution of Human Cytomegalovirus Is Shaped by Demography and Positive Selection
Source: PLoS Genet. 2013 Sep 26;9(9):e1003735. doi: 10.1371/journal.pgen.1003735 (PMC3784496; doi:10.1371/journal.pgen.1003735)
Supplement: Table S3 — Estimate of HCMV effective population size from time sampled populations. (PDF) [file pgen.1003735.s009.pdf]

Table S3: Estimate of HCMV Effective Population Size from Time Sampled Populations

Patient	Sample 1 Source	Sample 1 Time	Sample 2 Source	Sample 2 Time	Fs'[1]	N_e[2]
M103	Plasma	1.5 month	Plasma	5 month	1.08E-03	926
B103	Plasma	1 week	Plasma	6 month	1.31E-03	762
B101	Urine	7 month	Urine	10 month	2.09E-03	478
B103	Urine	1 week	Urine	6 month	1.26E-03	795
MS1	Urine	1 month	Urine	2 month	7.35E-04	1360
MS1	Urine	2 month	Urine	11 month	1.20E-03	831
MS2	Urine	1 month	Urine	2 month	6.90E-04	1450
MS2	Urine	2 month	Urine	11 month	9.19E-04	1088

1. Fs' is an estimator of SNP frequency variance
2. Effective population size
