# Supplementary material for: Rapid Intrahost Evolution of Human Cytomegalovirus Is Shaped by Demography and Positive Selection
Source: PLoS Genet. 2013 Sep 26;9(9):e1003735. doi: 10.1371/journal.pgen.1003735 (PMC3784496; doi:10.1371/journal.pgen.1003735)
Supplement: Table S4 — Targets of positive selection in 6 month B103 plasma populations. (PDF) [file pgen.1003735.s010.pdf]

Table S4: Targets of Positive Selection in 6 month B103 Plasma Populations

Feature	Type	Position	Frequency (1 week)	Frequency (6 months)	Fst	PBS	Coding	Syn/Non	AA Change
UL7	gene	16409	0.03	1.00	0.95	2.14	Yes	Non	S182L
UL7	gene	16410	0.01	0.99	0.97	2.35	Yes	Syn	
UL7	gene	16468	0.01	1.00	0.99	2.45	Yes	Non	L202I
UL7	gene	16476	0.06	0.97	0.89	1.75	Yes	Syn	
UL13	gene	20189	0.04	0.99	0.98	1.86	Yes	Non	A178T
Whole Genome	noncoding	22649	0.75	1.00	0.71	1.76	No	---	
UL20	gene	26154	0.78	1.00	0.97	2.18	Yes	Non	V172A
UL20	gene	26362	0.77	1.00	0.99	3.09	Yes	Syn	
UL32	gene	43303	0.50	1.00	0.79	1.74	Yes	Syn	
UL45	gene	60001	0.22	1.00	0.99	2.06	Yes	Syn	
UL45	gene	60043	0.21	1.00	0.98	2.11	Yes	Syn	
UL45	gene	60277	0.23	1.00	0.99	2.93	Yes	Syn	
Whole Genome	noncoding	87813	0.33	1.00	0.91	1.78	No	---	
UL73	gene	107285	0.02	0.99	0.97	2.45	Yes	Non	H79N
UL74	gene	108020	0.27	1.00	0.76	1.76	Yes	Non	S277T
UL74	gene	108183	0.23	1.00	0.81	1.82	Yes	Syn	
UL80	gene	117592	0.03	1.00	0.97	1.95	Yes	Syn	
UL80.5	gene	117592	0.03	1.00	0.97	1.95	Yes	Syn	
UL83	gene	121000	0.00	0.92	1.00	2.27	Yes	Non	S448A
UL116	gene	166563	0.34	1.00	0.74	1.74	Yes	Syn	
UL117	gene	166985	0.34	1.00	0.73	1.74	Yes	Syn	
US14	gene	208719	0.06	1.00	0.93	1.95	Yes	Non	T252K
Whole Genome	noncoding	221594	0.02	1.00	0.96	2.20	No	---	
