# Supplementary material for: Rapid Intrahost Evolution of Human Cytomegalovirus Is Shaped by Demography and Positive Selection
Source: PLoS Genet. 2013 Sep 26;9(9):e1003735. doi: 10.1371/journal.pgen.1003735 (PMC3784496; doi:10.1371/journal.pgen.1003735)
Supplement: Table S5 — Targets of positive selection in 6 month B103 urine populations. (PDF) [file pgen.1003735.s011.pdf]

Table S5: Targets of Positive Selection in 6 month B103 Urine Populations

Feature	Type	Position	Frequency (1 week)	Frequency (6 months)	Fst	PBS	Coding	Syn/Non	AA Change
TRS1	gene	233642	0.00	1.00	1.00	2.73	Yes	Non	T373G
TRS1	gene	233648	0.00	1.00	1.00	2.82	Yes	Non	G371L
