# Supplementary material for: Rapid Intrahost Evolution of Human Cytomegalovirus Is Shaped by Demography and Positive Selection
Source: PLoS Genet. 2013 Sep 26;9(9):e1003735. doi: 10.1371/journal.pgen.1003735 (PMC3784496; doi:10.1371/journal.pgen.1003735)
Supplement: Table S6 — Targets of positive selection in 10 month B101 urine populations. (PDF) [file pgen.1003735.s012.pdf]

Table S6: Targets of Positive Selection in 10 month B101 Urine Populations

Feature	Type	Position	Frequency (7 months)	Frequency (10 months)	Fst (B101C-B101D)	PBS	Coding	Syn/Non	AA Change
RL10	gene	8666	0.00	1.00	1.00	1.73	Yes	Syn	
Whole Genome	noncoding	13727	0.01	1.00	1.00	1.86	No	---	
Whole Genome	noncoding	13734	0.00	1.00	1.00	1.64	No	---	
UL4	gene	14174	0.25	1.00	0.80	1.81	Yes	Syn	
UL7	gene	15952	0.09	1.00	0.99	2.28	Yes	Non	T30A
UL7	gene	16411	0.00	1.00	1.00	1.80	Yes	Non	N183H
UL7	gene	16459	0.10	0.99	0.87	1.85	Yes		
UL7	gene	16461	0.07	1.00	0.90	1.96	Yes		
UL7	gene	16476	0.10	1.00	0.88	1.92	Yes	Syn	
UL73	gene	107156	0.03	1.00	0.09	1.65	Yes	Non	P36A
Whole Genome	noncoding	213424	0.17	1.00	0.93	1.64	No	---	
