# Supplementary material for: Rapid Intrahost Evolution of Human Cytomegalovirus Is Shaped by Demography and Positive Selection
Source: PLoS Genet. 2013 Sep 26;9(9):e1003735. doi: 10.1371/journal.pgen.1003735 (PMC3784496; doi:10.1371/journal.pgen.1003735)
Supplement: Table S7 — Targets of positive selection in 5 month M103 plasma populations. (PDF) [file pgen.1003735.s013.pdf]

Table S7: Targets of Positive Selection in 5 month M103 Plasma Populations

Feature	Type	Position	Frequency (1.5 months)	Frequency (5 months)	Fst	PBS	Coding	Syn/Non	AA Change
RL1	gene	1654	0.00	0.96	1.00	2.32	Yes	Syn	
UL13	gene	19816	0.00	0.96	0.99	2.28	Yes	Syn	
UL48	gene	68002	0.13	1.00	0.99	2.09	Yes	Syn	
