# Supplementary material for: Rapid Intrahost Evolution of Human Cytomegalovirus Is Shaped by Demography and Positive Selection
Source: PLoS Genet. 2013 Sep 26;9(9):e1003735. doi: 10.1371/journal.pgen.1003735 (PMC3784496; doi:10.1371/journal.pgen.1003735)
Supplement: Table S8 — Targets of positive selection in MS1 1 month urine populations. (PDF) [file pgen.1003735.s014.pdf]

Table S8: Targets of Positive Selection in MS1 1 month Urine Populations

Feature	Type	Position	Frequency (MS2)	Frequency (MS1)	Fst	PBS	Coding	Syn/Non	AA Change
UL48	gene	69346	0.00	1.00	1.00	2.61	Yes	Syn	
UL48	gene	69376	0.00	1.00	1.00	2.80	Yes	Syn	
UL48	gene	69430	0.00	1.00	1.00	2.10	Yes	Syn	
UL48	gene	69437	0.00	1.00	1.00	2.16	Yes	Non	Y1605H
UL48	gene	69499	0.00	0.99	0.99	1.91	Yes	Syn	
UL48	gene	69640	0.00	0.99	0.99	2.06	Yes	Syn	
UL48	gene	69694	0.00	1.00	1.00	2.50	Yes	Syn	
UL48	gene	69796	0.00	0.99	0.99	2.18	Yes	Syn	
UL50	gene	74129	0.03	0.92	0.92	2.05	Yes	Syn	
Whole Genome	noncoding	95699	0.00	1.00	1.00	2.90	No	---	
Whole Genome	noncoding	95700	0.00	1.00	1.00	2.19	No	---	
UL86	gene	127086	0.00	1.00	1.00	2.33	Yes	Syn	
UL148	gene	179584	0.00	1.00	1.00	2.71	Yes	Non	T170A
UL148	gene	179963	0.00	1.00	1.00	2.48	Yes	Syn	
UL148	gene	180008	0.00	1.00	1.00	2.96	Yes	Syn	
UL147A	gene	180316	0.00	1.00	1.00	3.18	Yes	Syn	
UL147A	gene	180349	0.00	1.00	1.00	3.03	Yes	Syn	
UL147A	gene	180352	0.00	1.00	1.00	2.94	Yes	Syn	
UL147A	gene	180361	0.00	1.00	1.00	3.17	Yes	Syn	
UL147	gene	180401	0.01	1.00	1.00	2.96	Yes	Syn	
UL147	gene	180405	0.01	1.00	1.00	2.95	Yes	Syn	
UL147	gene	180417	0.00	1.00	1.00	3.24	Yes	Syn	
UL147	gene	180492	0.00	1.00	1.00	2.62	Yes	Syn	
