# Supplementary material for: Rapid Intrahost Evolution of Human Cytomegalovirus Is Shaped by Demography and Positive Selection
Source: PLoS Genet. 2013 Sep 26;9(9):e1003735. doi: 10.1371/journal.pgen.1003735 (PMC3784496; doi:10.1371/journal.pgen.1003735)
Supplement: Table S9 — Targets of positive selection in MS2 1 month urine populations. (PDF) [file pgen.1003735.s015.pdf]

Table S9: Targets of Positive Selection in MS2 1 month Urine Populations

Feature	Type	Position	Frequency (MS1)	Frequency (MS2)	Fst	PBS	Coding	Syn/Non	AA Change
Whole Genome	noncoding	22216	0.25	1.00	0.96	2.34	No	---	
UL48	gene	69787	0.00	1.00	0.99	2.36	Yes	Syn	
UL56	gene	84820	0.03	0.96	0.93	2.09	Yes	Non	S829A
UL56	gene	84821	0.01	0.87	0.95	2.07	Yes	Syn	
UL86	gene	125820	0.00	1.00	1.00	3.12	Yes	Syn	
UL86	gene	127800	0.00	1.00	1.00	2.67	Yes	Syn	
UL86	gene	128196	0.00	1.00	1.00	2.35	Yes	Syn	
UL86	gene	128793	0.00	1.00	1.00	2.81	Yes	Syn	
UL86	gene	129216	0.00	1.00	1.00	2.81	Yes	Syn	
UL86	gene	129306	0.00	1.00	1.00	2.46	Yes	Syn	
UL86	gene	129318	0.00	1.00	1.00	2.66	Yes	Syn	
UL87	gene	132468	0.00	1.00	1.00	2.89	Yes	Non	A936V
Whole Genome	noncoding	230399	0.25	1.00	0.98	1.78	No	---	
