# Supplementary material for: Rapid Intrahost Evolution of Human Cytomegalovirus Is Shaped by Demography and Positive Selection
Source: PLoS Genet. 2013 Sep 26;9(9):e1003735. doi: 10.1371/journal.pgen.1003735 (PMC3784496; doi:10.1371/journal.pgen.1003735)
Supplement: Table S10 — Targets of positive selection in MS1 2 month urine populations. (PDF) [file pgen.1003735.s016.pdf]

Table S10: Targets of Positive Selection in MS1 2 month Urine Populations

Feature	Type	Position	Frequency (MS2)	Frequency (MS1)	Fst	PBS	Coding	Syn/Non	AA Change
Whole Genome	noncoding	190764	0.00	1.00	1.00	2.98	No	---	
UL148A	gene	190872	0.00	1.00	1.00	2.40	Yes	Syn	
UL148A	gene	190905	0.00	1.00	1.00	3.17	Yes	Syn	
UL148A	gene	191019	0.00	1.00	1.00	2.31	Yes	Syn	
UL148C	gene	191725	0.00	1.00	1.00	3.03	Yes	Syn	
UL148D	gene	192166	0.00	1.00	1.00	2.96	Yes	Syn	
UL148D	gene	192192	0.00	1.00	1.00	2.72	Yes	Non	Q19R
UL148D	gene	192294	0.00	1.00	1.00	2.90	Yes	Non	Q53R
UL148D	gene	192298	0.00	1.00	1.00	3.00	Yes	Syn	
