# Supplementary material for: Rapid Intrahost Evolution of Human Cytomegalovirus Is Shaped by Demography and Positive Selection
Source: PLoS Genet. 2013 Sep 26;9(9):e1003735. doi: 10.1371/journal.pgen.1003735 (PMC3784496; doi:10.1371/journal.pgen.1003735)
Supplement: Table S11 — Targets of positive selection in MS2 2 month urine populations. (PDF) [file pgen.1003735.s017.pdf]

Table S11: Targets of Positive Selection in MS2 2 month Urine Populations

Feature	Type	Position	Frequency (MS1)	Frequency (MS2)	Fst	PBS	Coding	Syn/Non	AA Change
UL55	gene	83375	0.00	1.00	1.00	2.83	Yes	Syn	
UL82	gene	120206	0.00	1.00	1.00	3.05	Yes	Syn	
Whole Genome	noncoding	120468	0.00	1.00	1.00	2.53	No	---	
Whole Genome	noncoding	120594	0.07	1.00	0.98	2.36	No	---	
UL83	gene	120665	0.04	1.00	0.98	1.84	Yes	Syn	
UL83	gene	120703	0.09	1.00	0.96	1.82	Yes	Syn	
UL83	gene	121060	0.00	1.00	1.00	2.75	Yes	Non	G428S
UL83	gene	121097	0.00	1.00	1.00	2.56	Yes	Syn	
UL83	gene	121276	0.00	1.00	1.00	2.66	Yes	Syn	
