# Supplementary material for: Rapid Intrahost Evolution of Human Cytomegalovirus Is Shaped by Demography and Positive Selection
Source: PLoS Genet. 2013 Sep 26;9(9):e1003735. doi: 10.1371/journal.pgen.1003735 (PMC3784496; doi:10.1371/journal.pgen.1003735)
Supplement: Table S12 — Targets of positive selection in MS1 11 month urine populations. (PDF) [file pgen.1003735.s018.pdf]

Table S12: Targets of Positive Selection in MS1 11 month Urine Populations

Feature	Type	Position	Frequency (MS2)	Frequency (MS1)	Fst	PBS	Coding	Syn/Non	AA Change
UL76	gene	112213	0.00	0.97	1.00	2.56	Yes	Syn	
UL132	gene	178699	0.00	1.00	1.00	2.42	Yes	Syn	
UL132	gene	178723	0.00	1.00	1.00	2.50	Yes	Syn	
UL132	gene	178761	0.00	1.00	1.00	2.64	Yes	Non	V102I
UL132	gene	178934	0.00	1.00	1.00	2.53	Yes	Non	N44S
UL132	gene	178954	0.00	1.00	1.00	2.98	Yes	Non	K37N
UL132	gene	178971	0.00	1.00	1.00	3.00	Yes	Non	V32M
UL132	gene	178996	0.00	1.00	1.00	2.75	Yes	Non	Q23H
UL132	gene	179024	0.00	1.00	1.00	2.23	Yes	Non	A14V
UL132	gene	179045	0.00	1.00	1.00	2.59	Yes	Non	P7L
UL148C	gene	191725	0.00	1.00	1.00	3.00	Yes	Syn	
