# Supplementary material for: Rapid Intrahost Evolution of Human Cytomegalovirus Is Shaped by Demography and Positive Selection
Source: PLoS Genet. 2013 Sep 26;9(9):e1003735. doi: 10.1371/journal.pgen.1003735 (PMC3784496; doi:10.1371/journal.pgen.1003735)
Supplement: Table S13 — Targets of positive selection in MS2 11 month urine populations. (PDF) [file pgen.1003735.s019.pdf]

Table S13: Targets of Positive Selection in MS2 11 month Urine Populations

Feature	Type	Position	Frequency (MS1)	Frequency (MS2)	Fst	PBS	Coding	Syn/Non	AA Change
UL77	gene	113336	0.04	1.00	0.95	1.95	Yes	Syn	
UL77	gene	113339	0.05	1.00	0.93	1.88	Yes	Syn	
UL77	gene	113672	0.06	1.00	0.95	2.12	Yes	Syn	
UL77	gene	113675	0.06	1.00	0.89	1.97	Yes	Syn	
UL77	gene	113681	0.16	1.00	0.75	1.78	Yes	Syn	
UL119	gene	168225	0.00	1.00	1.00	3.00	Yes	Syn	
