# Supplementary material for: Rapid Intrahost Evolution of Human Cytomegalovirus Is Shaped by Demography and Positive Selection
Source: PLoS Genet. 2013 Sep 26;9(9):e1003735. doi: 10.1371/journal.pgen.1003735 (PMC3784496; doi:10.1371/journal.pgen.1003735)
Supplement: Table S14 — Targets of positive selection in 1 week B103 urine populations. (PDF) [file pgen.1003735.s020.pdf]

Table S14: Targets of Positive Selection in 1 week B103 Urine Populations

Feature	Type	Position	Frequency (Plasma)	Frequency (Urine)	Fst	PBS	Coding	Syn/Non	AA Change
RL1	gene	1452	0.00	0.97	1.00	2.65	Yes	Non	R29T
RL1	gene	1628	0.00	0.87	0.99	2.50	Yes	Non	R88C
Whole Genome	noncoding	2336	0.00	1.00	1.00	2.56	No	---	
RL5A	gene	5630	0.00	1.00	1.00	2.65	Yes	Non	T16I
RL5A	gene	5667	0.00	1.00	1.00	2.65	Yes	Non	R4G
Whole Genome	noncoding	6460	0.00	1.00	1.00	3.00	No	---	
Whole Genome	noncoding	6474	0.00	1.00	1.00	3.00	No	---	
Whole Genome	noncoding	6718	0.00	1.00	1.00	3.00	No	---	
Whole Genome	noncoding	6733	0.00	1.00	1.00	3.00	No	---	
Whole Genome	noncoding	7609	0.00	1.00	1.00	2.76	No	---	
Whole Genome	noncoding	7813	0.00	1.00	1.00	2.76	No	---	
Whole Genome	noncoding	7833	0.00	1.00	1.00	3.00	No	---	
Whole Genome	noncoding	7837	0.00	1.00	1.00	3.00	No	---	
Whole Genome	noncoding	8068	0.00	1.00	1.00	2.73	No	---	
Whole Genome	noncoding	8441	0.00	1.00	1.00	3.00	No	---	
RL10	gene	8720	0.00	1.00	1.00	3.00	Yes	Syn	
RL10	gene	8726	0.00	1.00	1.00	3.00	Yes	Syn	
Whole Genome	noncoding	9114	0.00	1.00	1.00	2.76	No	---	
RL11	gene	9267	0.00	1.00	1.00	3.00	Yes	Syn	
RL11	gene	9291	0.00	1.00	1.00	2.85	Yes	Syn	
RL11	gene	9294	0.00	1.00	1.00	2.98	Yes	Syn	
UL20	gene	25958	0.00	1.00	1.00	3.00	Yes	Non	D107Q
UL20	gene	25990	0.00	1.00	1.00	3.00	Yes	Syn	

UL23	gene	28536	0.00	1.00	1.00	2.70	Yes	Non	S176T
UL37	gene	50646	0.00	1.00	1.00	3.00	Yes	Syn	
UL37	gene	51123	0.00	1.00	1.00	3.00	Yes	Syn	
UL44	gene	57542	0.00	1.00	1.00	2.65	Yes	Syn	
UL45	gene	58123	0.00	1.00	1.00	3.00	Yes	Syn	
UL48	gene	67687	0.00	1.00	1.00	2.85	Yes	Syn	
UL48	gene	67985	0.00	1.00	1.00	3.00	Yes	Non	I1121V
UL48	gene	68101	0.00	1.00	1.00	2.76	Yes	Syn	
UL48	gene	68107	0.00	1.00	1.00	2.65	Yes	Syn	
UL55	gene	82953	0.01	1.00	1.00	2.64	Yes	Non	L613F
UL55	gene	83215	0.01	1.00	0.99	2.55	Yes	Syn	
UL55	gene	83375	0.00	1.00	1.00	2.46	Yes	Non	S472N
UL55	gene	83527	0.00	1.00	1.00	3.00	Yes	Syn	
UL55	gene	83722	0.00	1.00	1.00	3.00	Yes	Syn	
UL55	gene	83872	0.01	1.00	1.00	2.61	Yes	Syn	
UL55	gene	83992	0.10	1.00	0.99	2.46	Yes	Syn	
UL55	gene	84295	0.00	1.00	1.00	2.58	Yes	Syn	
UL55	gene	84358	0.00	1.00	1.00	2.46	Yes	Syn	
UL55	gene	84424	0.00	1.00	1.00	2.50	Yes	Syn	
UL56	gene	85418	0.00	1.00	1.00	2.43	Yes	Syn	
UL56	gene	85664	0.00	1.00	1.00	2.76	Yes	Syn	
Whole Genome	noncoding	93489	0.00	1.00	1.00	3.00	No	---	
Whole Genome	noncoding	93490	0.00	1.00	1.00	3.00	No	---	
UL82	gene	119994	0.00	0.99	1.00	2.50	Yes	Non	E158G
UL82	gene	119999	0.00	0.99	1.00	2.67	Yes	Non	M157V
UL82	gene	120206	0.00	1.00	1.00	2.70	Yes	Syn	
UL83	gene	121352	0.00	1.00	1.00	2.70	Yes	Syn	
UL83	gene	121385	0.00	1.00	1.00	2.99	Yes	Syn	
UL83	gene	121790	0.00	1.00	1.00	2.76	Yes	Syn	
UL86	gene	126066	0.00	1.00	1.00	2.99	Yes	Syn	
UL86	gene	126075	0.00	1.00	1.00	2.99	Yes	Syn	
UL86	gene	126091	0.00	0.98	1.00	3.00	Yes	Non	A1171V
UL86	gene	126192	0.00	1.00	1.00	2.46	Yes	Syn	
UL86	gene	126294	0.00	1.00	1.00	2.85	Yes	Syn	
UL86	gene	126999	0.00	1.00	1.00	2.52	Yes	Syn	

UL86	gene	127776	0.00	1.00	1.00	2.65	Yes	Syn	
UL86	gene	129480	0.00	1.00	1.00	3.00	Yes	Syn	
UL87	gene	130510	0.00	1.00	1.00	2.52	Yes	Syn	
UL104	gene	154843	0.00	0.98	1.00	2.52	Yes	Syn	
UL112	gene	162355	0.00	1.00	1.00	2.44	Yes	Non	S147G
UL112	gene	162412	0.01	1.00	1.00	3.00	Yes	Syn	
UL124	gene	174252	0.00	1.00	1.00	2.85	Yes	Syn	
UL124	gene	174306	0.00	1.00	1.00	2.44	Yes	Non	N62S
UL124	gene	174328	0.00	1.00	1.00	3.00	Yes	Non	R69H
Whole Genome	noncoding	174589	0.00	1.00	1.00	2.85	No	---	
Whole Genome	noncoding	174636	0.00	1.00	1.00	2.70	No	---	
Whole Genome	noncoding	174853	0.00	1.00	1.00	3.00	No	---	
Whole Genome	noncoding	174893	0.00	1.00	1.00	3.00	No	---	
Whole Genome	noncoding	174900	0.00	0.99	1.00	2.85	No	---	
Whole Genome	noncoding	175158	0.00	1.00	1.00	2.76	No	---	
Whole Genome	noncoding	175494	0.00	1.00	1.00	3.00	No	---	
Whole Genome	noncoding	175773	0.00	1.00	1.00	3.00	No	---	
UL148D	gene	192164	0.00	1.00	1.00	2.52	Yes	Non	T10A
US2	gene	199658	0.00	1.00	1.00	2.90	Yes	Syn	
US2	gene	199795	0.00	1.00	1.00	2.46	Yes	Non	Q46E
US3	gene	200703	0.00	1.00	1.00	3.00	Yes	Non	N68S
US3	gene	200748	0.00	0.99	1.00	2.55	Yes	Non	F53Y
US3	gene	200752	0.00	1.00	1.00	2.85	Yes	Non	Y52H
US3	gene	200753	0.00	1.00	1.00	2.60	Yes	Syn	
US3	gene	200869	0.00	1.00	1.00	2.85	Yes	Syn	
Whole Genome	noncoding	201156	0.00	1.00	1.00	2.54	No	---	
Whole Genome	noncoding	201320	0.00	1.00	1.00	2.76	No	---	
US6	gene	201624	0.00	1.00	1.00	2.76	Yes	Syn	

US6	gene	201713	0.00	1.00	1.00	3.00	Yes	Syn	
Whole Genome	noncoding	202372	0.00	1.00	1.00	3.00	No	---	
US7	gene	202688	0.00	1.00	1.00	3.00	Yes	Syn	
US7	gene	202716	0.00	1.00	1.00	2.85	Yes	Non	T184M
US7	gene	202772	0.00	1.00	1.00	2.50	Yes	Syn	
US7	gene	202819	0.00	0.92	1.00	2.98	Yes	Non	V150I
US7	gene	202907	0.00	1.00	1.00	3.00	Yes	Syn	
US7	gene	202914	0.00	1.00	1.00	3.00	Yes	Non	A118V
Whole Genome	noncoding	203349	0.00	1.00	1.00	2.76	No	---	
Whole Genome	noncoding	203398	0.00	1.00	1.00	2.65	No	---	
US12	gene	207365	0.17	1.00	1.00	2.58	Yes	Syn	
US14	gene	208721	0.00	1.00	1.00	3.00	Yes	Syn	
Whole Genome	noncoding	212491	0.00	1.00	1.00	3.00	No	---	
US21	gene	215482	0.00	1.00	1.00	2.85	Yes	Syn	
US22	gene	216387	0.00	1.00	1.00	2.50	Yes	Syn	
Whole Genome	noncoding	219773	0.01	1.00	0.99	2.43	No	---	
US27	gene	224755	0.00	1.00	1.00	2.60	Yes	Non	A208V
US27	gene	224885	0.00	1.00	1.00	3.00	Yes	Syn	
US27	gene	224916	0.00	1.00	1.00	2.85	Yes	Syn	
US28	gene	225485	0.00	1.00	1.00	2.97	Yes	Syn	
US29	gene	226815	0.04	1.00	1.00	2.73	Yes	Non	D52H
US29	gene	226853	0.11	1.00	1.00	2.58	Yes	Syn	
US30	gene	228636	0.00	1.00	1.00	2.55	Yes	Syn	
US31	gene	229101	0.00	1.00	1.00	2.85	Yes	Syn	
US32	gene	229974	0.00	1.00	1.00	3.00	Yes	Syn	
Whole Genome	noncoding	230236	0.00	1.00	1.00	2.85	No	---	
Whole Genome	noncoding	230282	0.00	1.00	1.00	2.69	No	---	
